# Supplementary figures and images for: A food-grade cell dissociation agent via regulatory pre-check framework
Source: PLoS One. 2026 Apr 13;21(4):e0345921. doi: 10.1371/journal.pone.0345921 (PMC13075713; doi:10.1371/journal.pone.0345921)

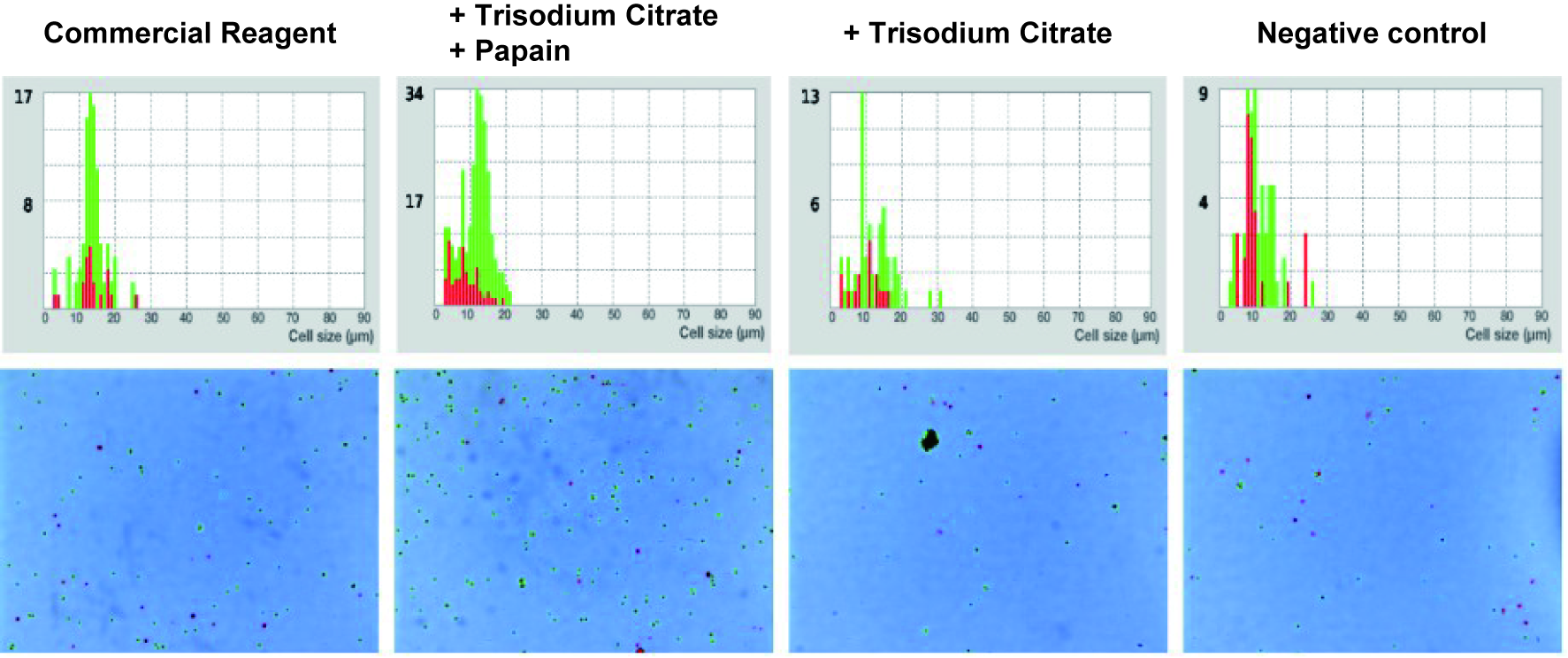

Supplement: S1 Fig — Green and red bars indicate live and dead cells, respectively. The brightfield images below each graph show the cell suspension used for the measurement. Observationally, the particle size distribution for the iDisper condition (+ Trisodium Citrate + Papain) showed a primary peak between 10–15 µm, similar to the Commercial Reagent. In contrast, the Trisodium Citrate alone and the Negative control conditions showed a main peak closer to 10 µm with a broader distribution, suggesting the presence of cell aggregates. These data are presented as a qualitative reference. (TIF) [file pone.0345921.s001.tif]

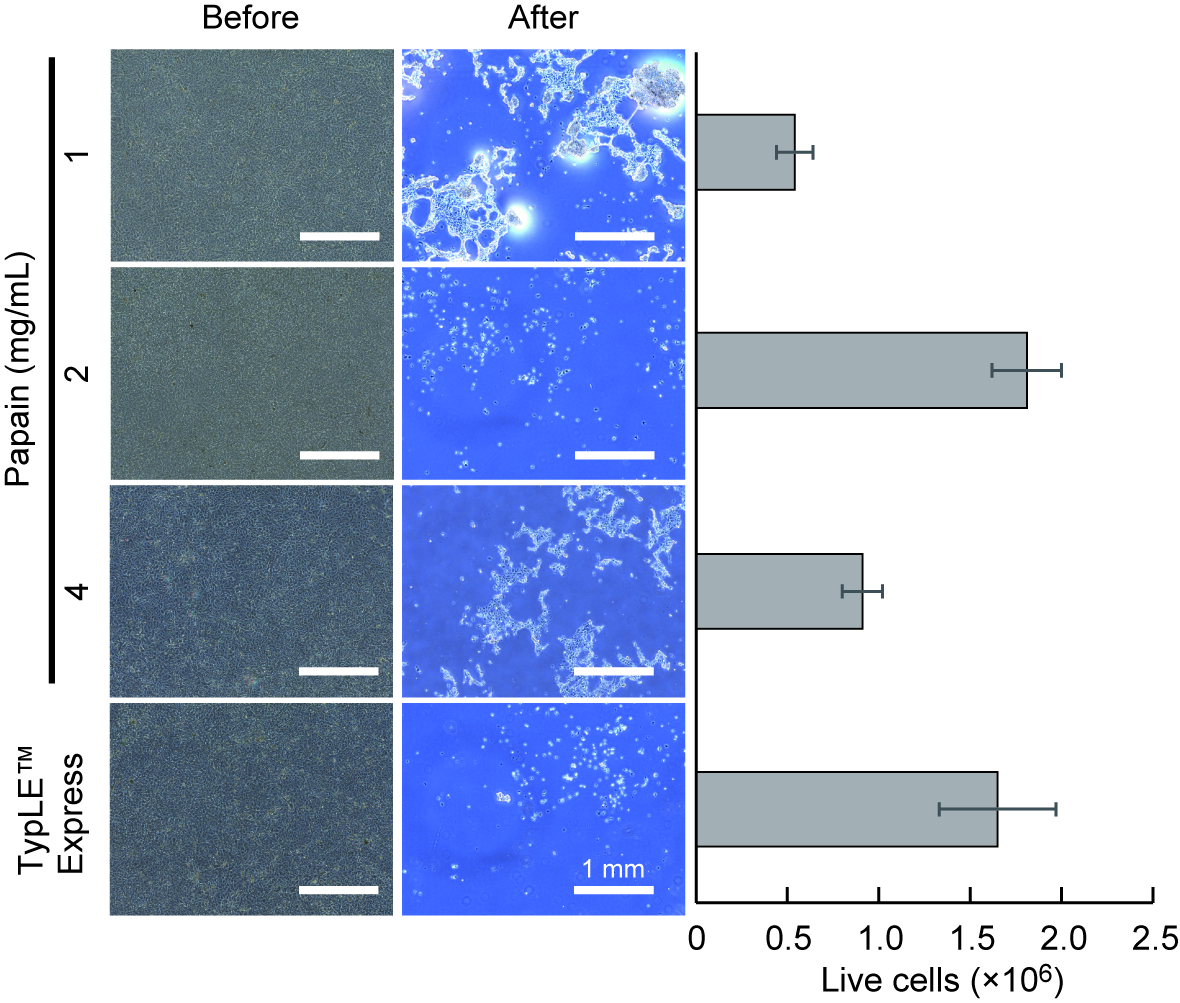

Supplement: S2 Fig — Phase-contrast microscopy images of primary duck liver cells before treatment (Before) and at 10 min after (After) the addition of iDisper containing papain at 1, 2, or 4 mg/mL. The control reagent, TrypLE™ Express, was treated for 5 min. The “After pipetting” column shows the plate surface after cell collection. All cultures were confluent before treatment. The bar chart shows the number of live cells recovered after detachment. Data are presented as mean ± SD (n = 3). Scale bar = 1 mm. (TIF) [file pone.0345921.s002.tif]

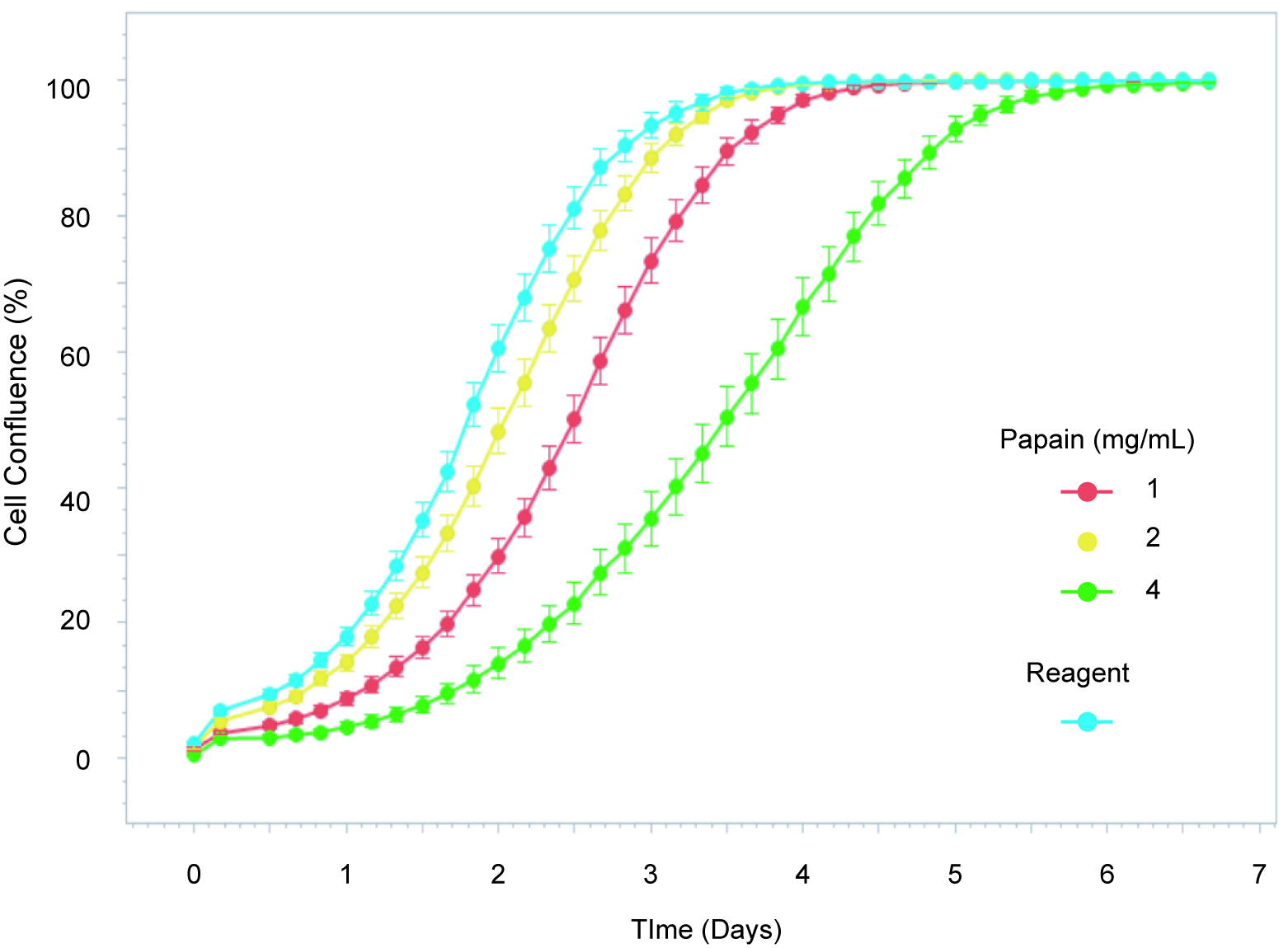

Supplement: S3 Fig — Growth curves of primary duck liver cells over approximately 7 days following detachment with iDisper containing papain at 1, 2, or 4 mg/mL, or with the control reagent TrypLE™ Express. Cell proliferation was evaluated by measuring cell confluence (%) over time using an IncuCyte® live-cell imaging and analysis system. Data are presented as mean ± SE (n = 3). (TIF) [file pone.0345921.s003.tif]

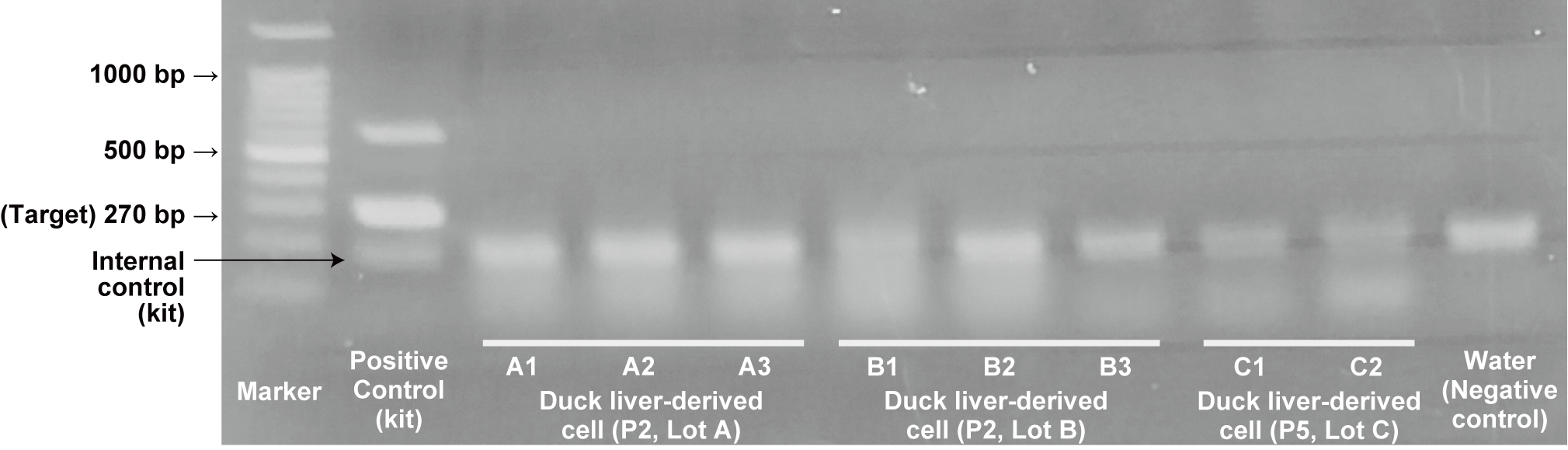

Supplement: S4 Fig — Duck liver cells passaged with iDisper were tested using the e-Myco™ plus Kit across three independent lots (Lots A, B, and C). Lanes: Marker: DNA ladder; Positive Control (kit): Positive control showing specific target bands (including 270 bp); Duck liver-derived cell (P2, Lot A): Cells at Passage 2 (n = 3); Duck liver-derived cell (P2, Lot B): Cells at Passage 2 (n = 3); Duck liver-derived cell (P5, Lot C): Cells at Passage 5 (n = 2); Water: Negative control. The presence of internal control bands in all sample lanes confirms successful PCR amplification, while the absence of the specific 270 bp band confirms that all cell cultures were free of mycoplasma contamination. (TIF) [file pone.0345921.s004.tif]

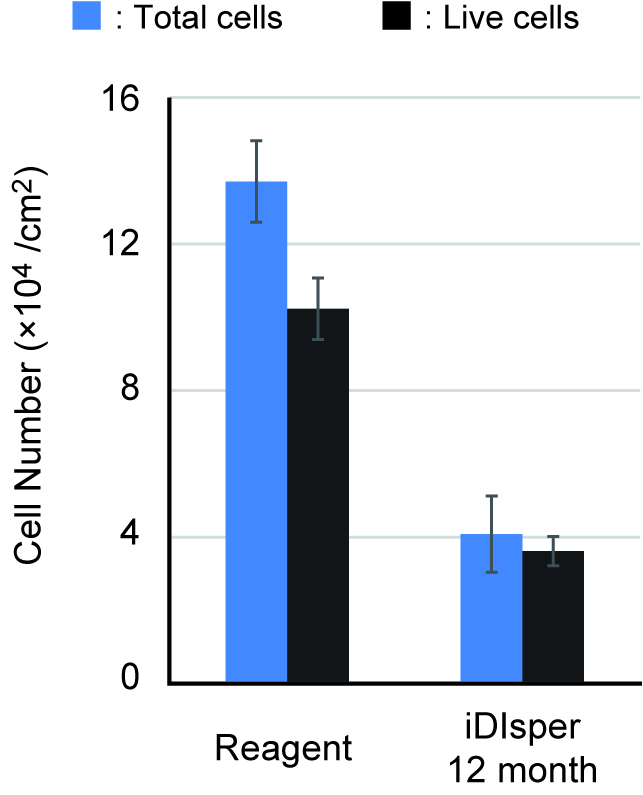

Supplement: S5 Fig — The cell detachment performance of iDisper stored for 12 months at －20°C was compared against that of a commercial reagent. Data are presented as mean ± SD (n = 3). The cell recovery rate after 12 months was below 50% of the control, which did not meet our internal criteria for further statistical analysis. (TIF) [file pone.0345921.s005.tif]

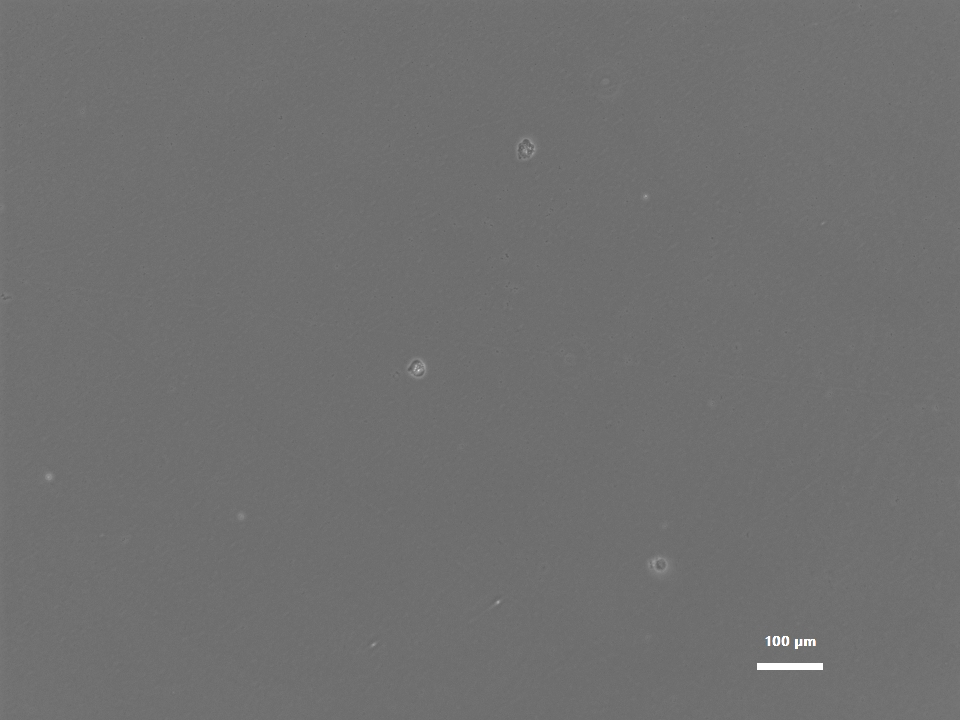

Supplement: S1 File — (ZIP) [file pone.0345921.s007.zip › Raw data for Fig2-4/Fig 3/Fig 3a +Trisodium citrate + papain after.png]

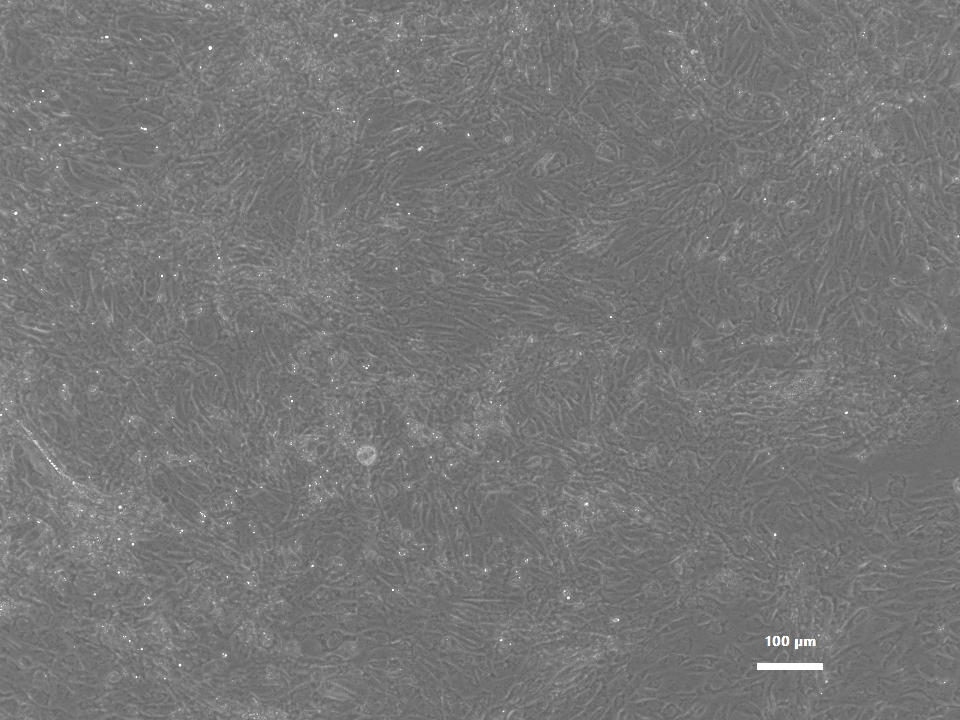

Supplement: S1 File — (ZIP) [file pone.0345921.s007.zip › Raw data for Fig2-4/Fig 3/Fig 3a +Trisodium citrate + papain before.png]

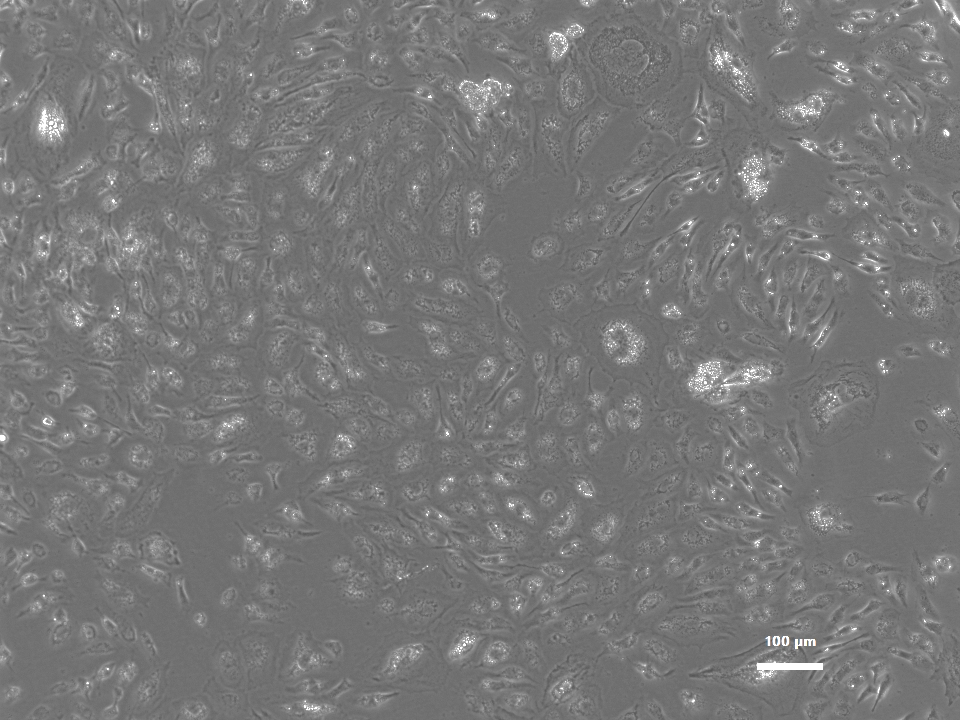

Supplement: S1 File — (ZIP) [file pone.0345921.s007.zip › Raw data for Fig2-4/Fig 3/Fig 3a +Trisodium citrate after.png]

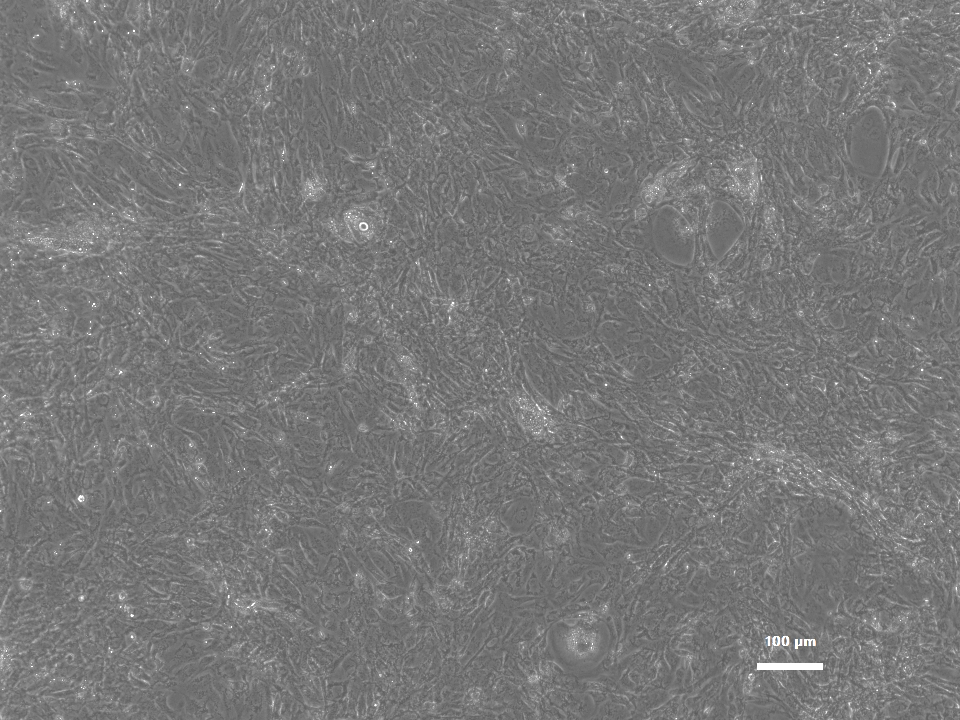

Supplement: S1 File — (ZIP) [file pone.0345921.s007.zip › Raw data for Fig2-4/Fig 3/Fig 3a +Trisodium citrate before.png]

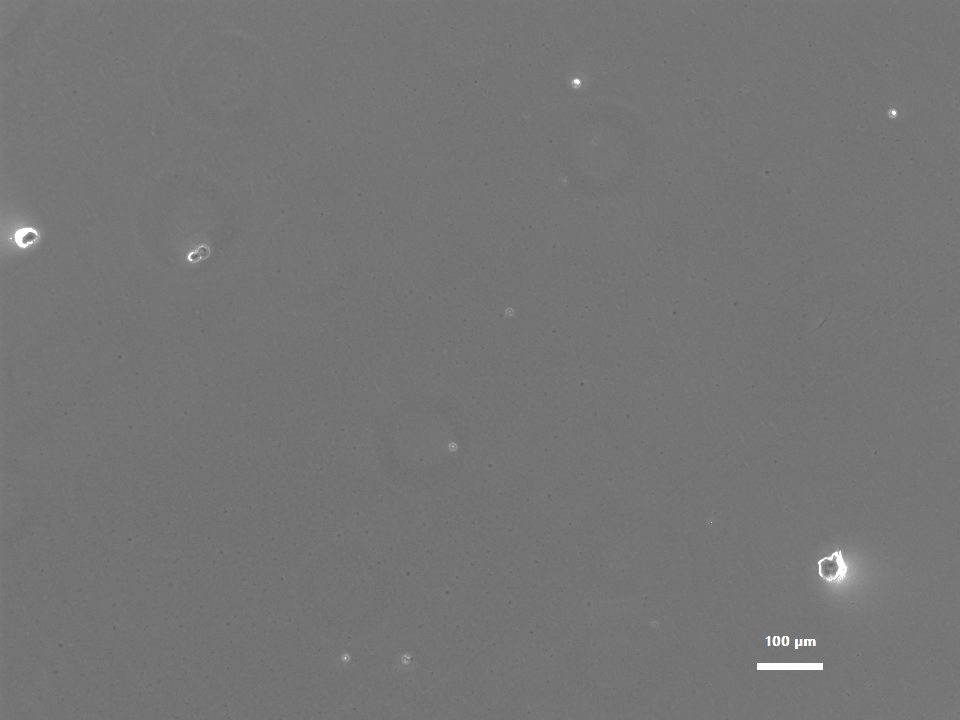

Supplement: S1 File — (ZIP) [file pone.0345921.s007.zip › Raw data for Fig2-4/Fig 3/Fig 3a commercial reagent after.png]

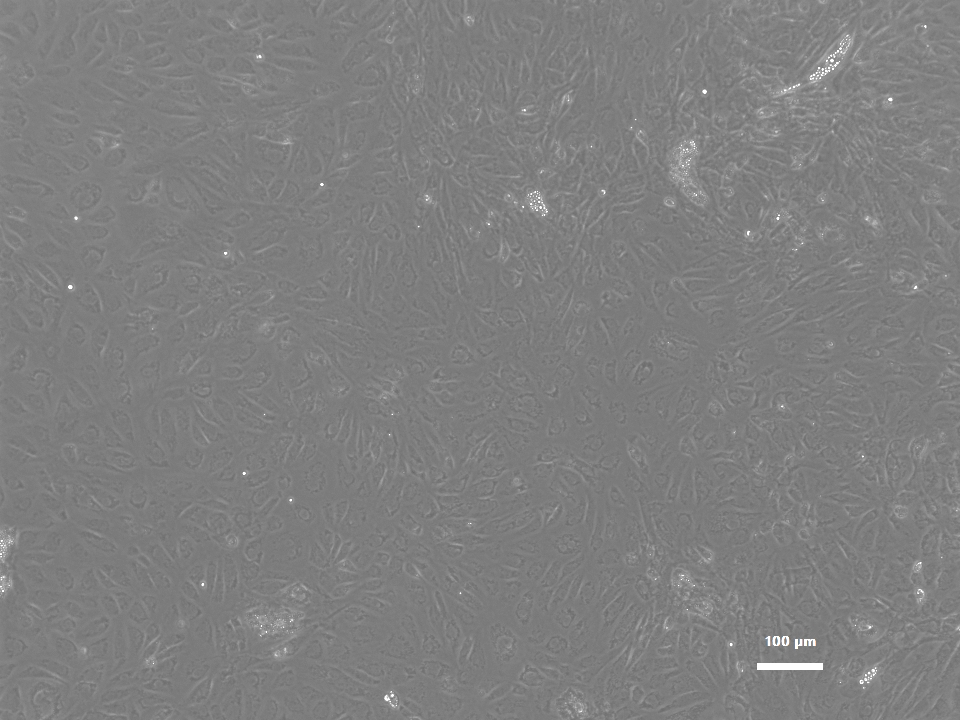

Supplement: S1 File — (ZIP) [file pone.0345921.s007.zip › Raw data for Fig2-4/Fig 3/Fig 3a commercial reagent before.png]

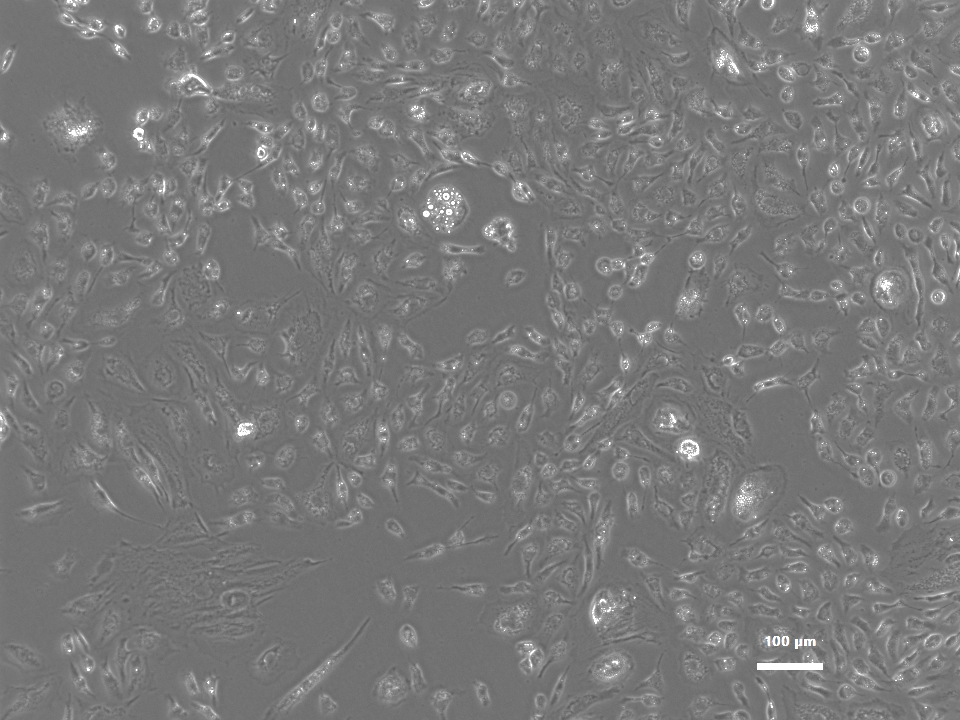

Supplement: S1 File — (ZIP) [file pone.0345921.s007.zip › Raw data for Fig2-4/Fig 3/Fig 3a negative control after.png]

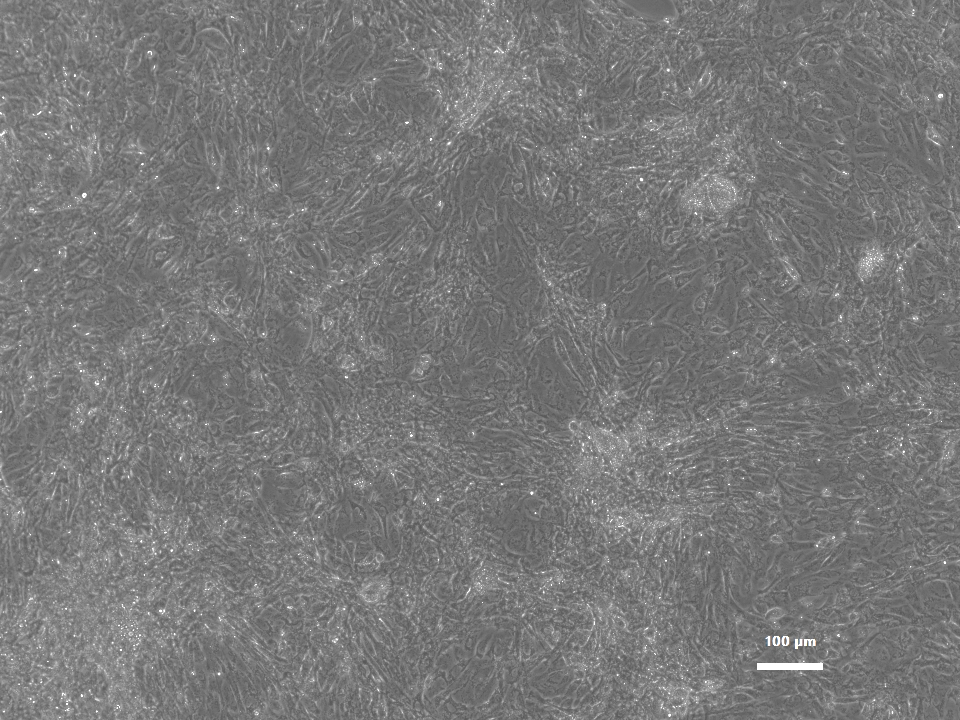

Supplement: S1 File — (ZIP) [file pone.0345921.s007.zip › Raw data for Fig2-4/Fig 3/Fig 3a negative control before.png]

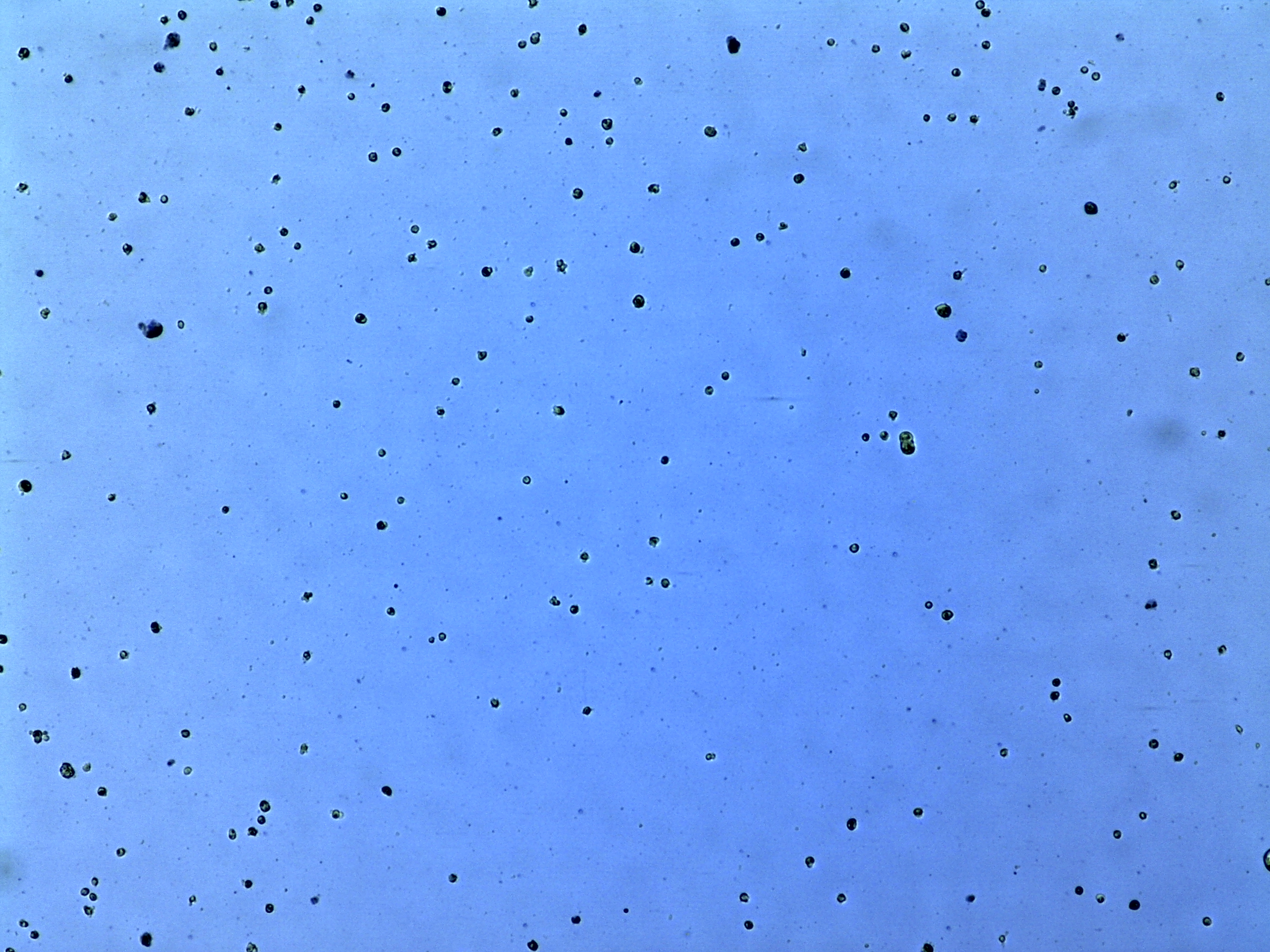

Supplement: S2 File — (ZIP) [file pone.0345921.s008.zip › Raw data for S1-5 Fig/S1 Fig/S1 Fig +Papain+Trisodium Citrate/hpc-5_RAW.tif πü«πé│πâöπüE.tif]

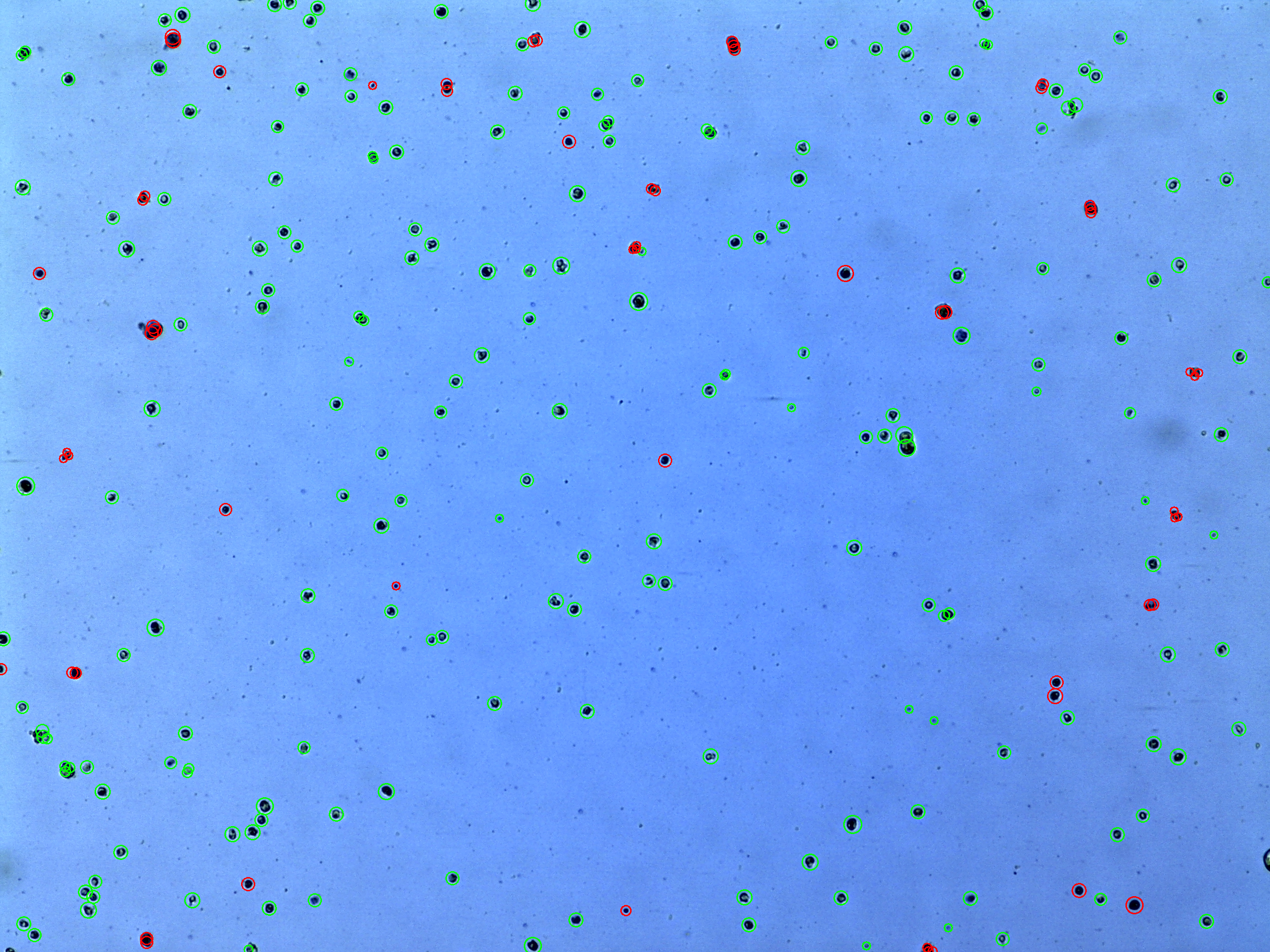

Supplement: S2 File — (ZIP) [file pone.0345921.s008.zip › Raw data for S1-5 Fig/S1 Fig/S1 Fig +Papain+Trisodium Citrate/hpc-5_TAG.tif πü«πé│πâöπüE.tif]

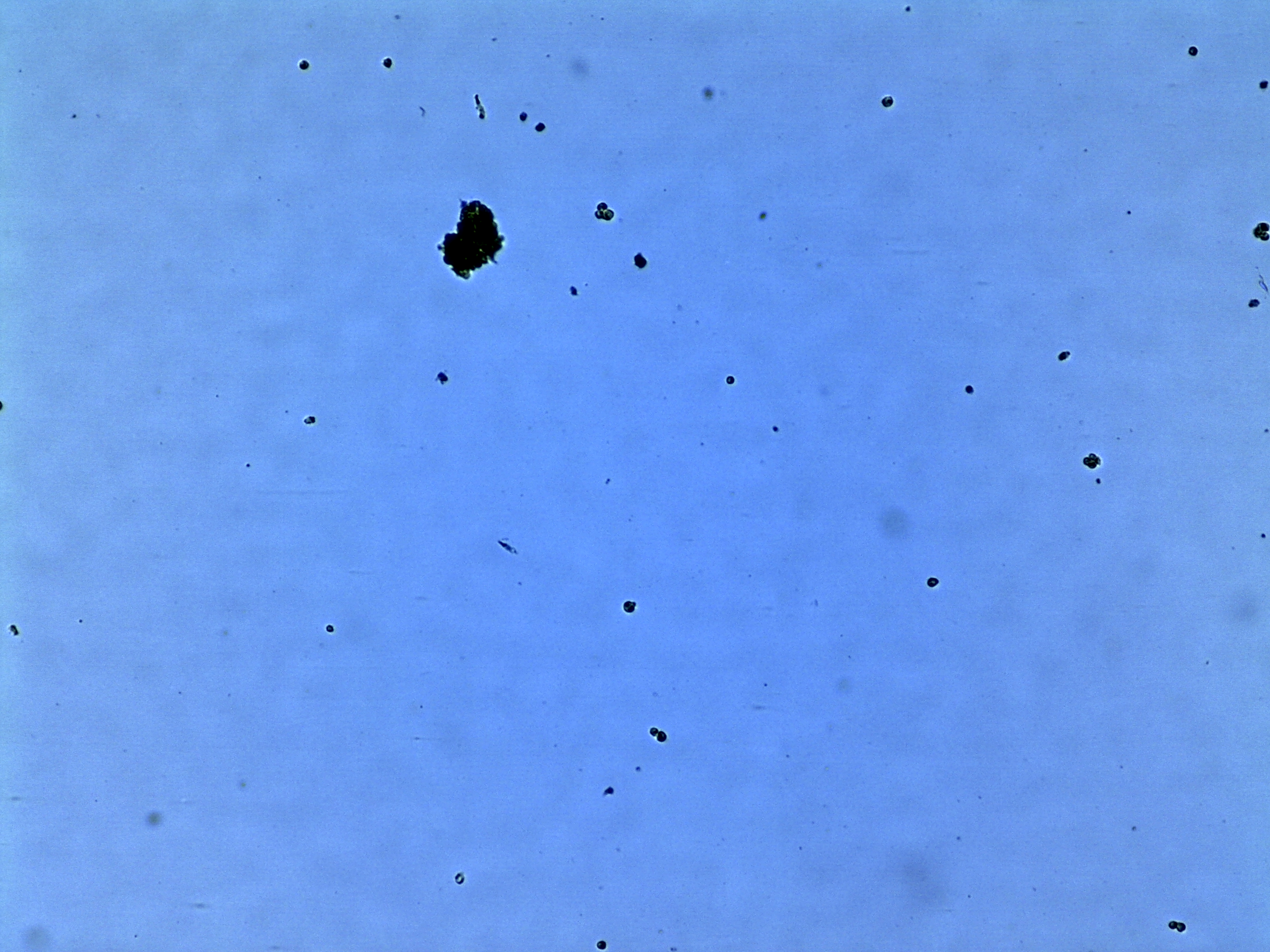

Supplement: S2 File — (ZIP) [file pone.0345921.s008.zip › Raw data for S1-5 Fig/S1 Fig/S1 Fig +Trisodium citrate/cirate10_RAW.tif πü«πé│πâöπüE.tif]

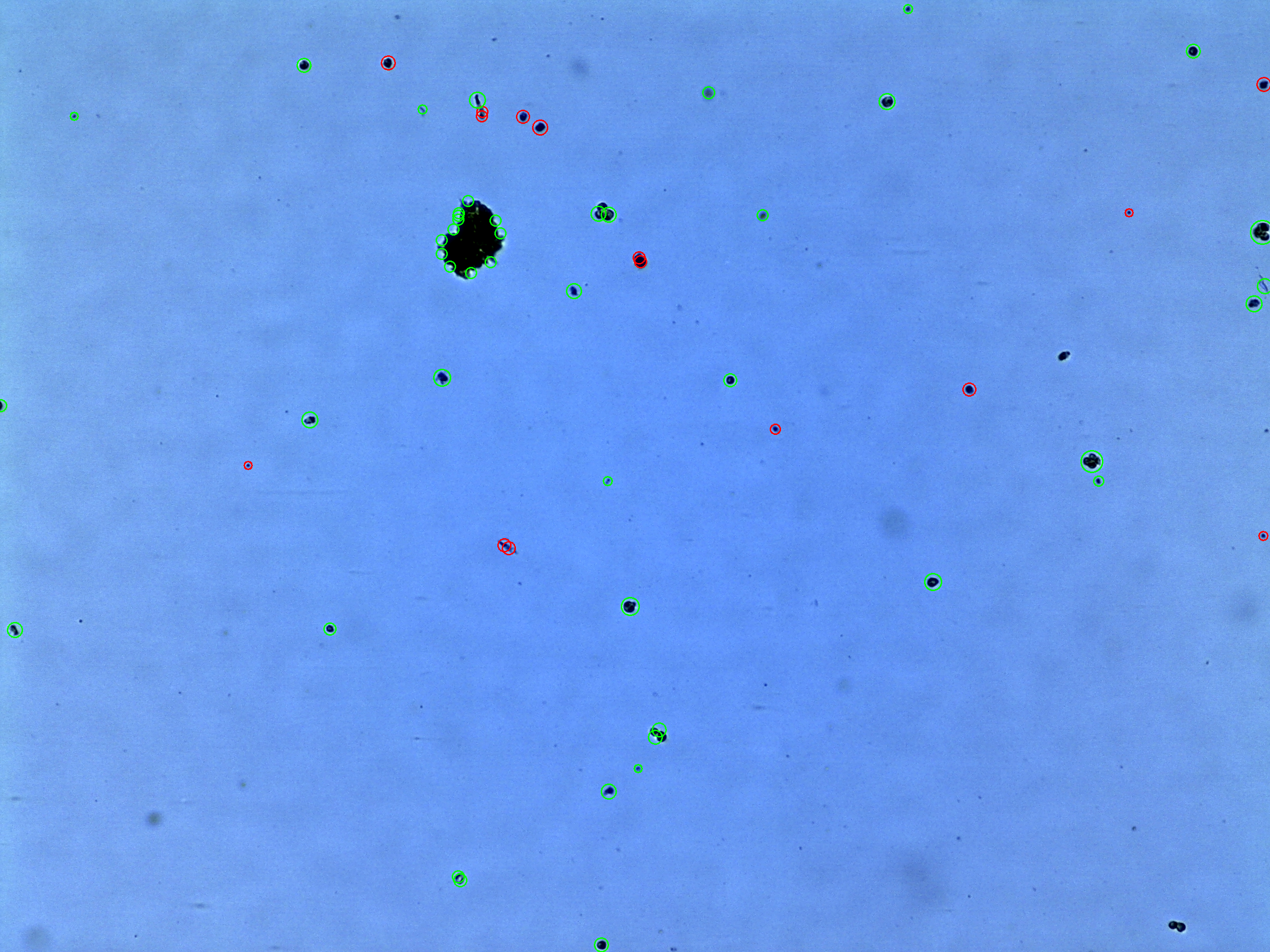

Supplement: S2 File — (ZIP) [file pone.0345921.s008.zip › Raw data for S1-5 Fig/S1 Fig/S1 Fig +Trisodium citrate/cirate10_TAG.tif πü«πé│πâöπüE.tif]

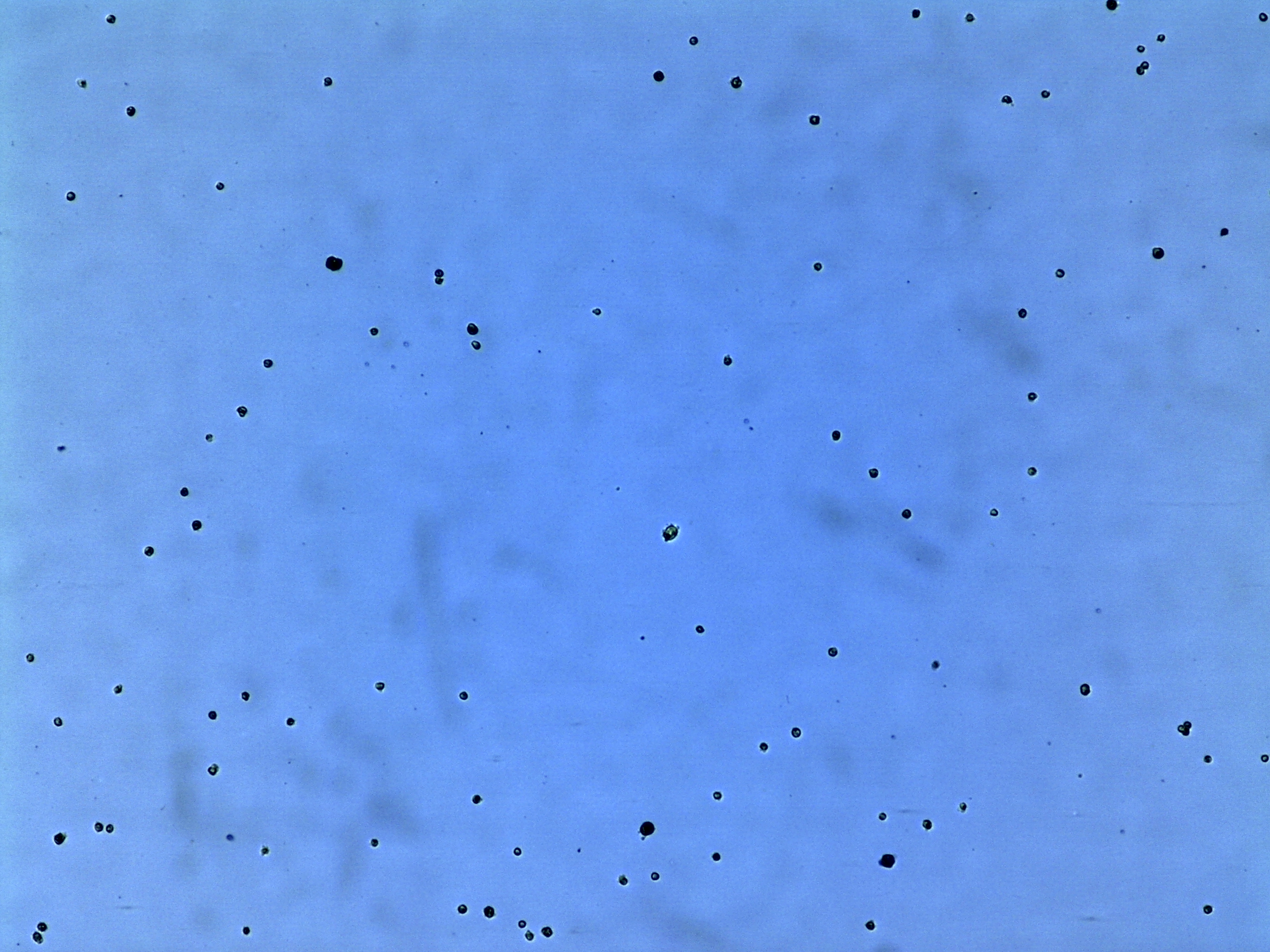

Supplement: S2 File — (ZIP) [file pone.0345921.s008.zip › Raw data for S1-5 Fig/S1 Fig/S1 Fig Commercial Reagent/tryple1_RAW.tif πü«πé│πâöπüE.tif]

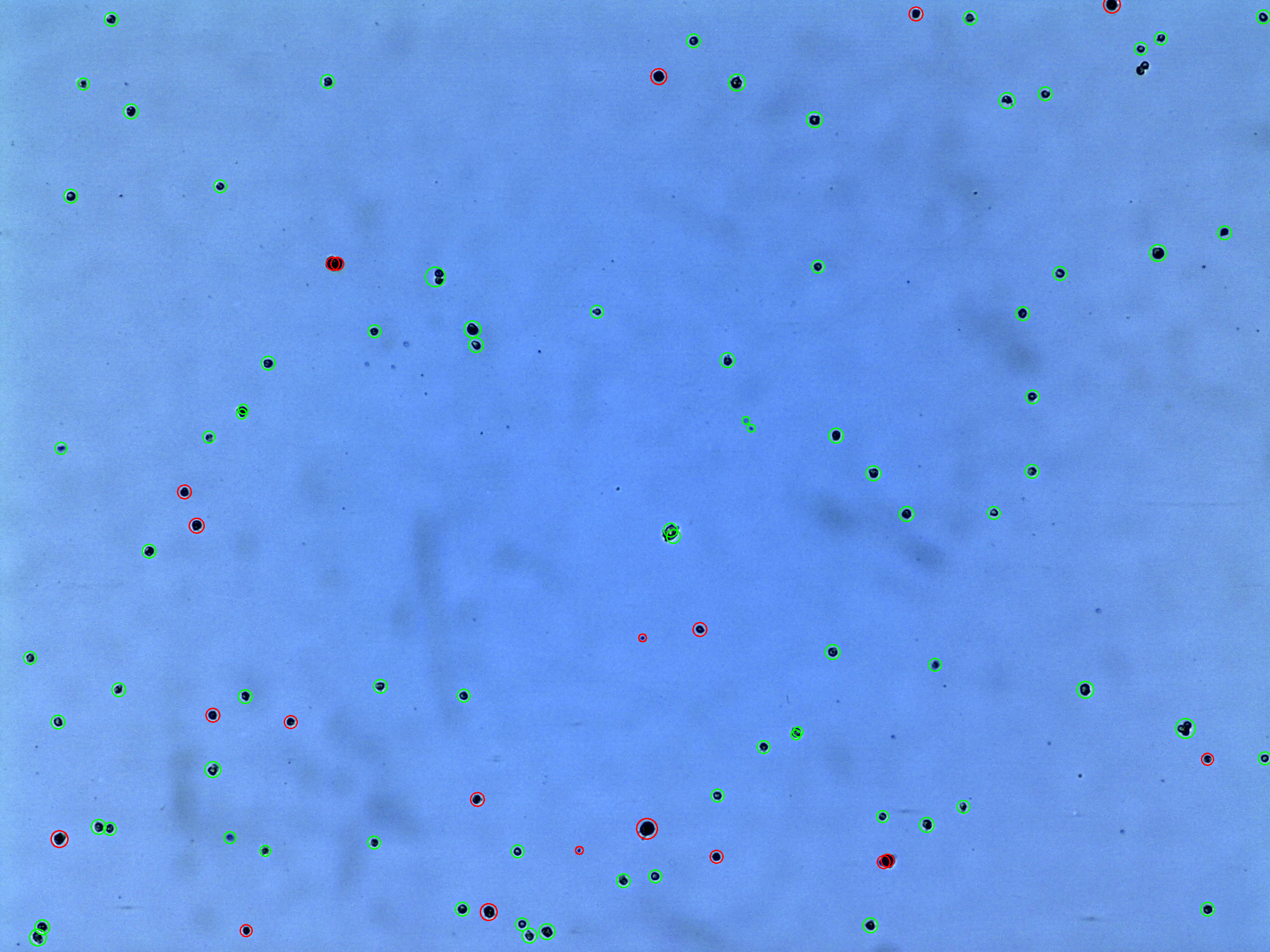

Supplement: S2 File — (ZIP) [file pone.0345921.s008.zip › Raw data for S1-5 Fig/S1 Fig/S1 Fig Commercial Reagent/tryple1_TAG.tif πü«πé│πâöπüE.tif]

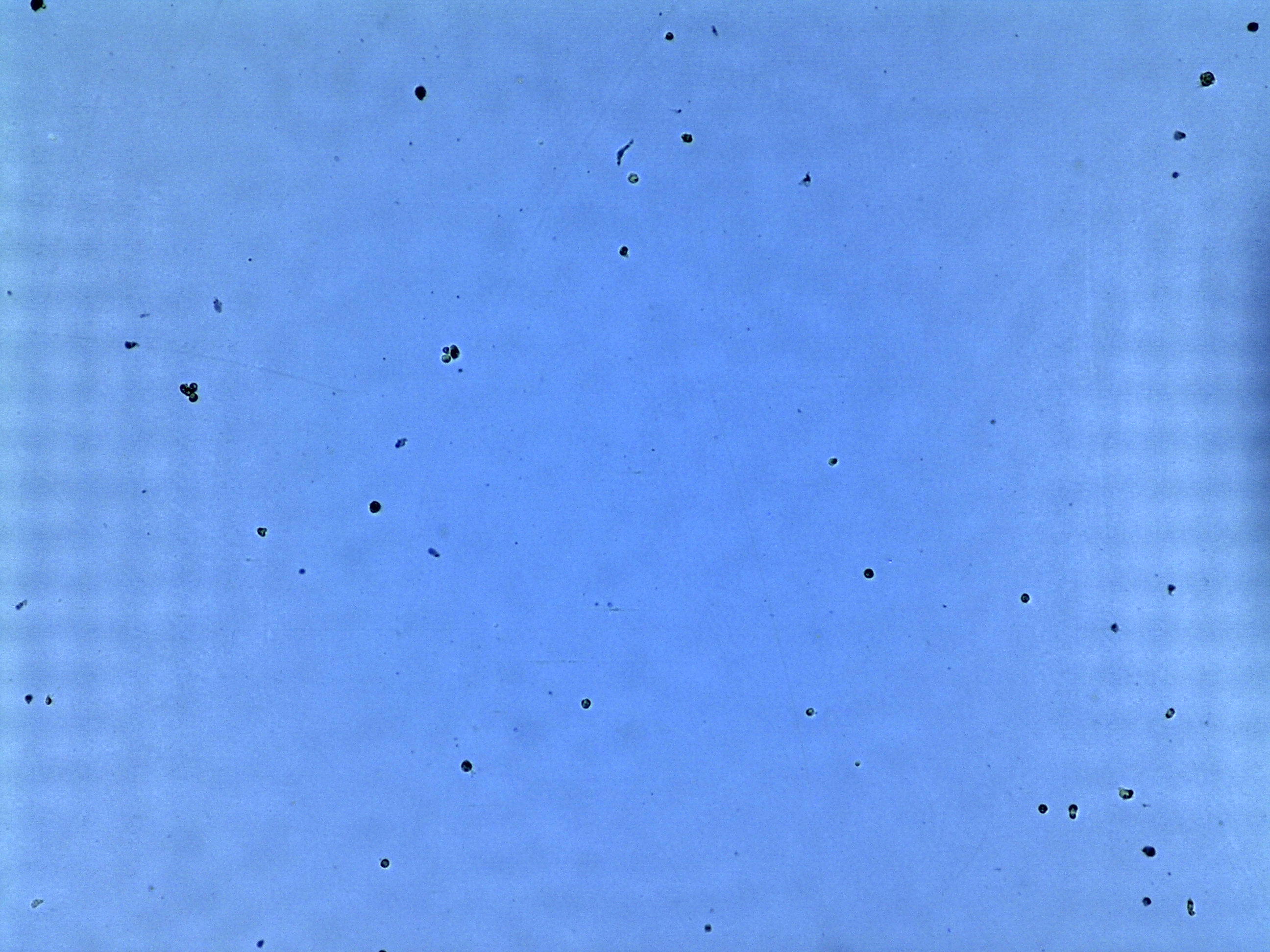

Supplement: S2 File — (ZIP) [file pone.0345921.s008.zip › Raw data for S1-5 Fig/S1 Fig/S1 Fig Negative control/ka13_RAW.tif πü«πé│πâöπüE.tif]

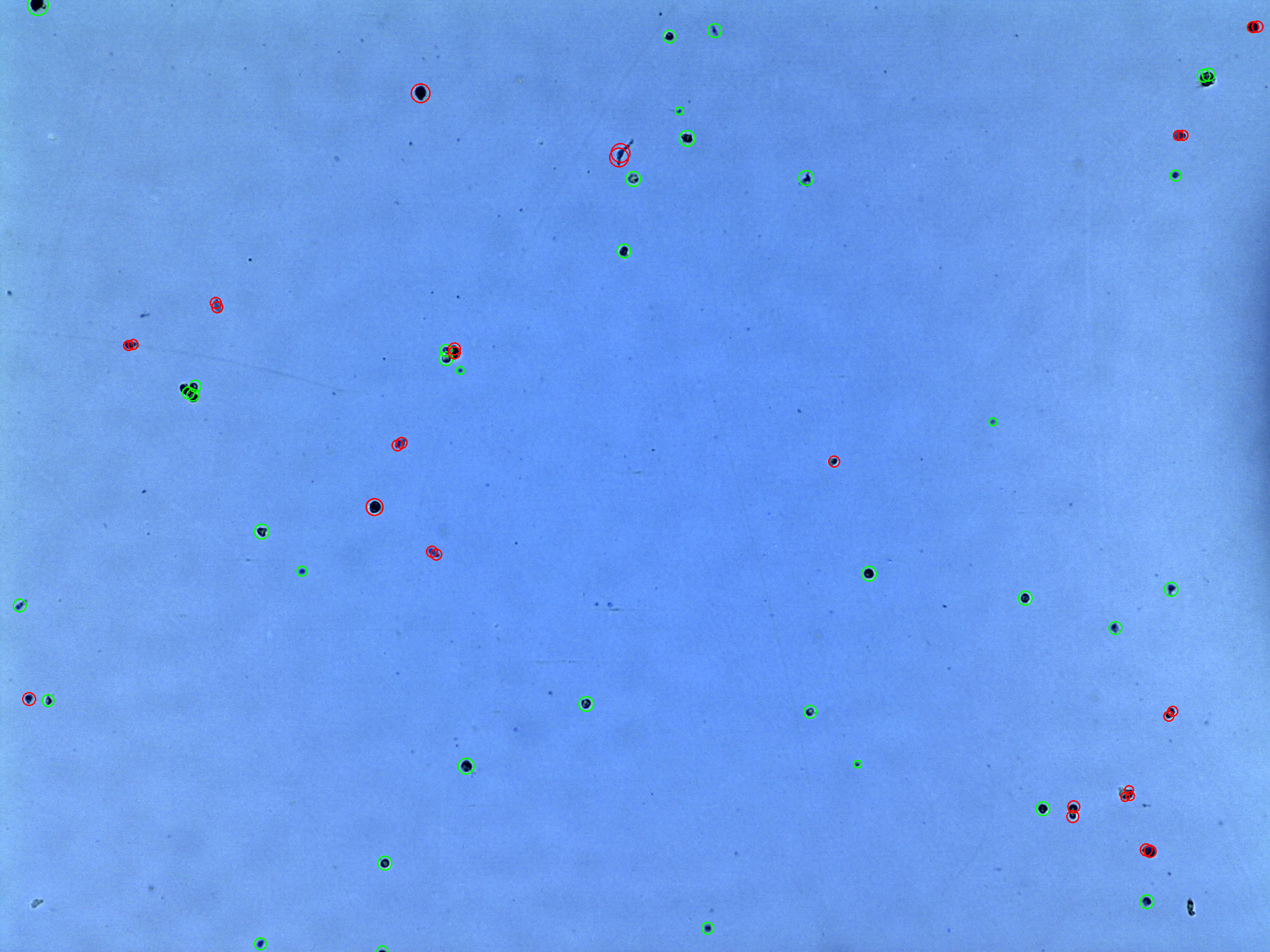

Supplement: S2 File — (ZIP) [file pone.0345921.s008.zip › Raw data for S1-5 Fig/S1 Fig/S1 Fig Negative control/ka13_TAG.tif πü«πé│πâöπüE.tif]

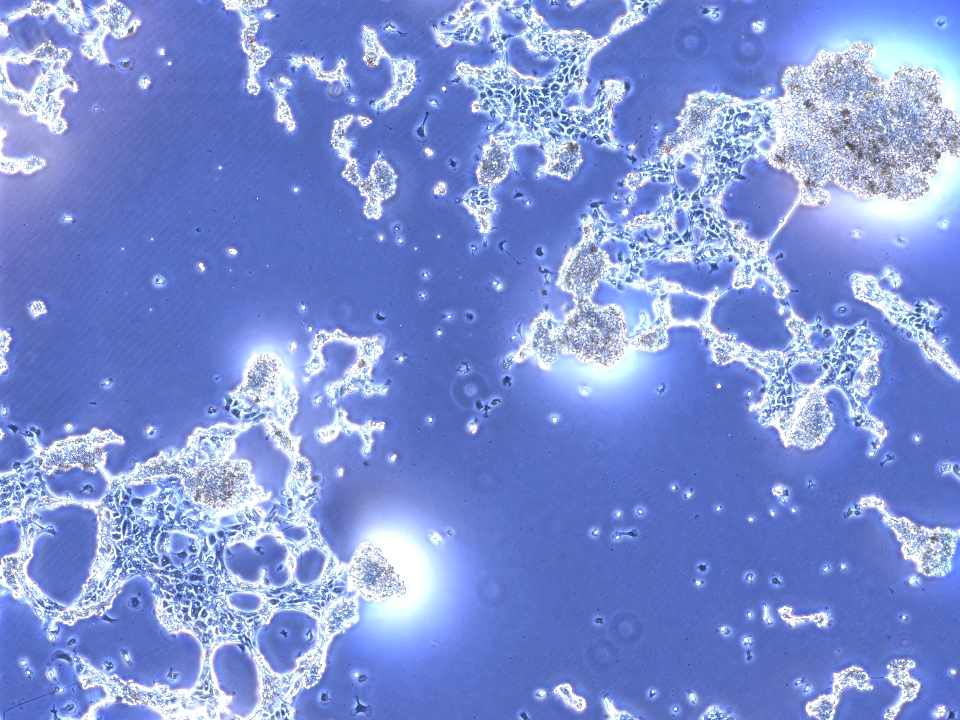

Supplement: S2 File — (ZIP) [file pone.0345921.s008.zip › Raw data for S1-5 Fig/S2 Fig/1 mgmL papain after.jpg]

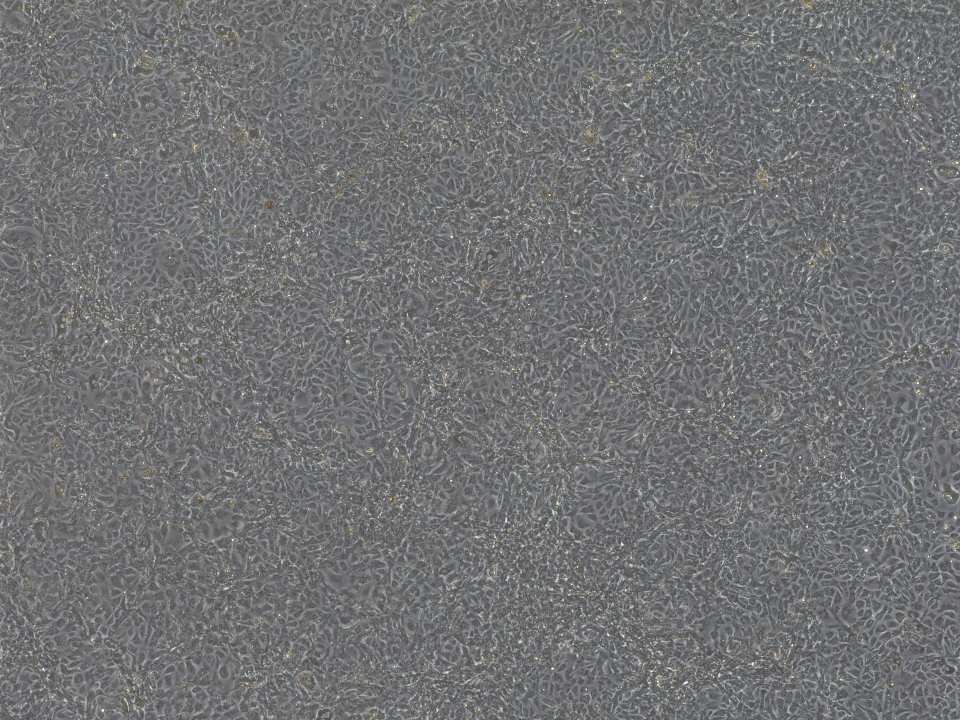

Supplement: S2 File — (ZIP) [file pone.0345921.s008.zip › Raw data for S1-5 Fig/S2 Fig/1 mgmL papain before.jpg]

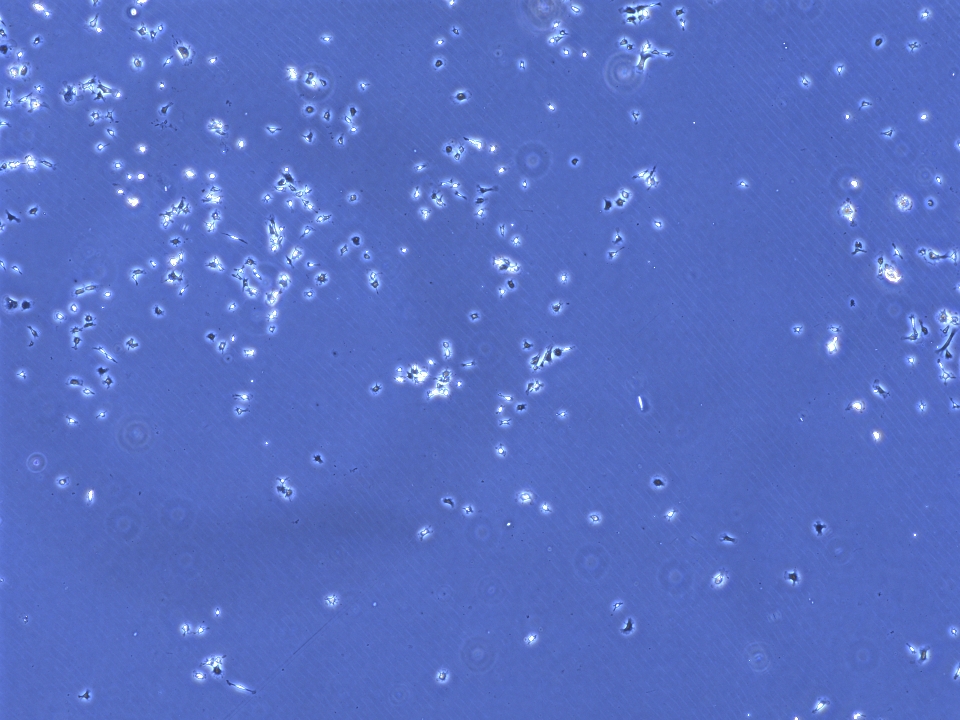

Supplement: S2 File — (ZIP) [file pone.0345921.s008.zip › Raw data for S1-5 Fig/S2 Fig/2 mgmL papain after.jpg]

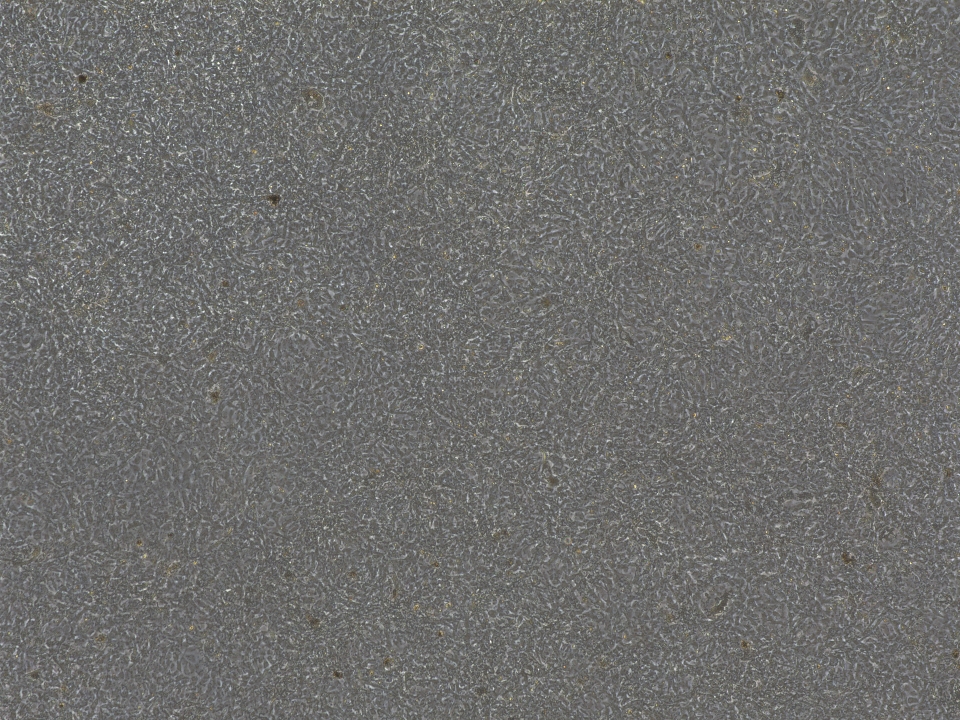

Supplement: S2 File — (ZIP) [file pone.0345921.s008.zip › Raw data for S1-5 Fig/S2 Fig/2 mgmL papain before.jpg]

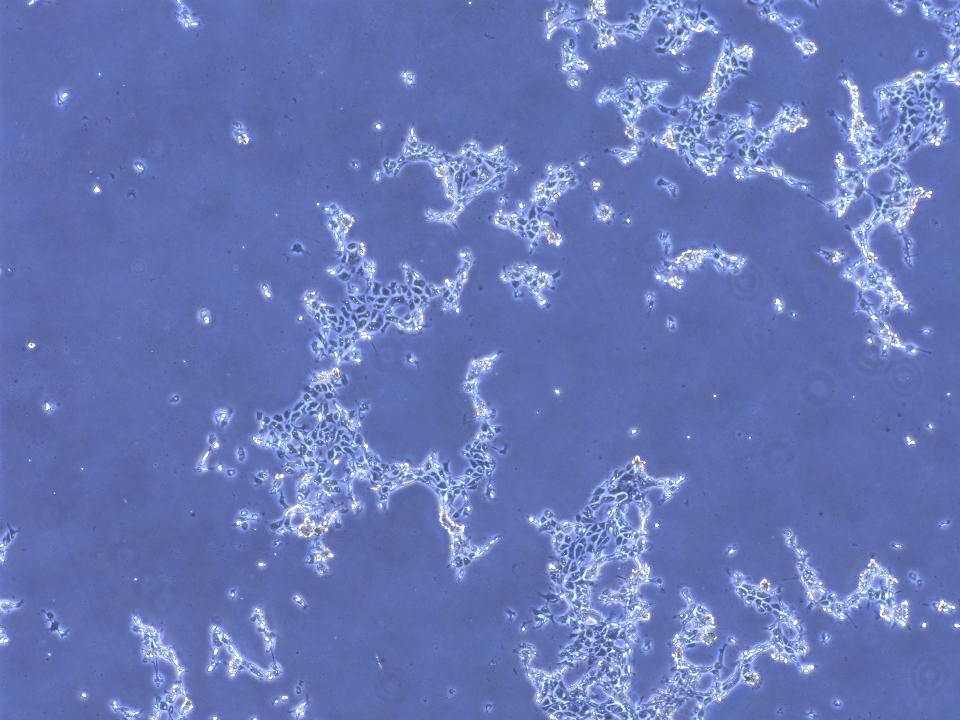

Supplement: S2 File — (ZIP) [file pone.0345921.s008.zip › Raw data for S1-5 Fig/S2 Fig/4 mgmL papain after.jpg]

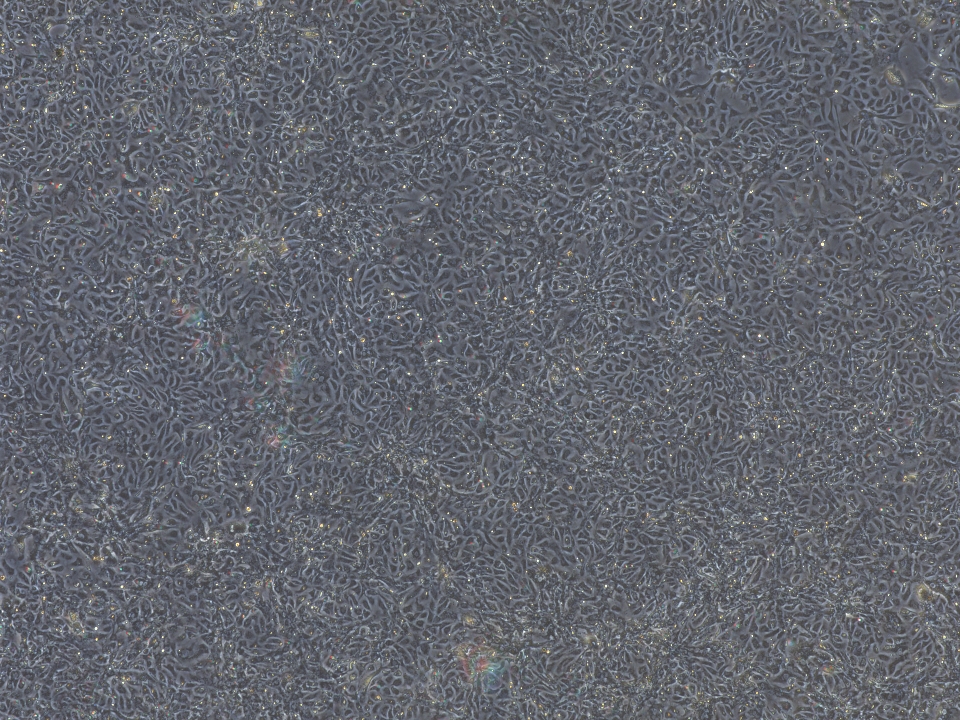

Supplement: S2 File — (ZIP) [file pone.0345921.s008.zip › Raw data for S1-5 Fig/S2 Fig/4 mgmL papain before.jpg]

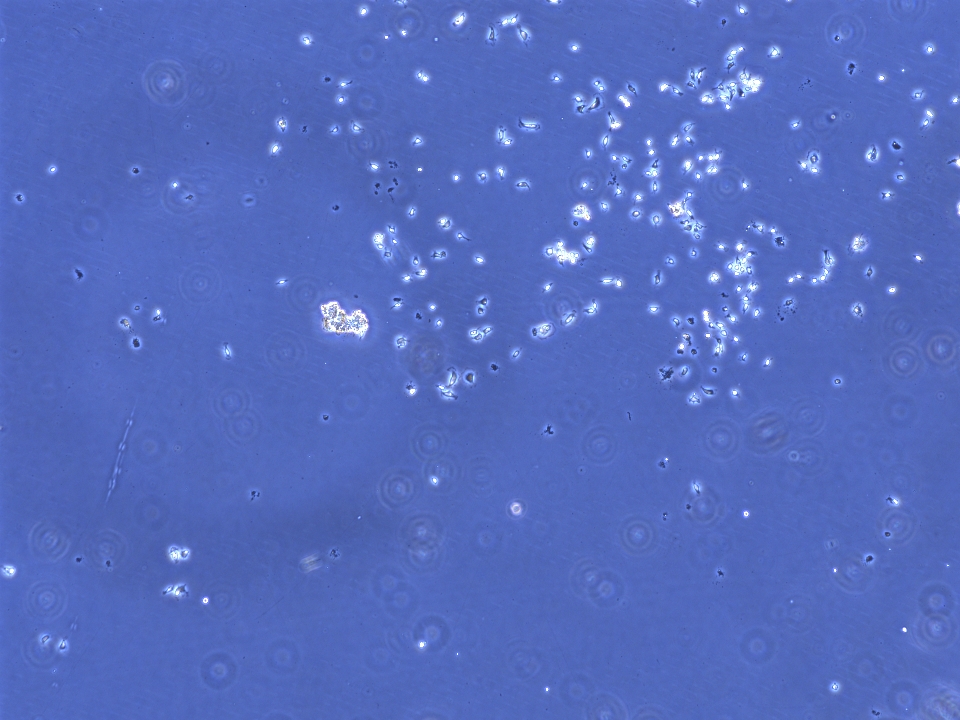

Supplement: S2 File — (ZIP) [file pone.0345921.s008.zip › Raw data for S1-5 Fig/S2 Fig/TrypLE after.jpg]

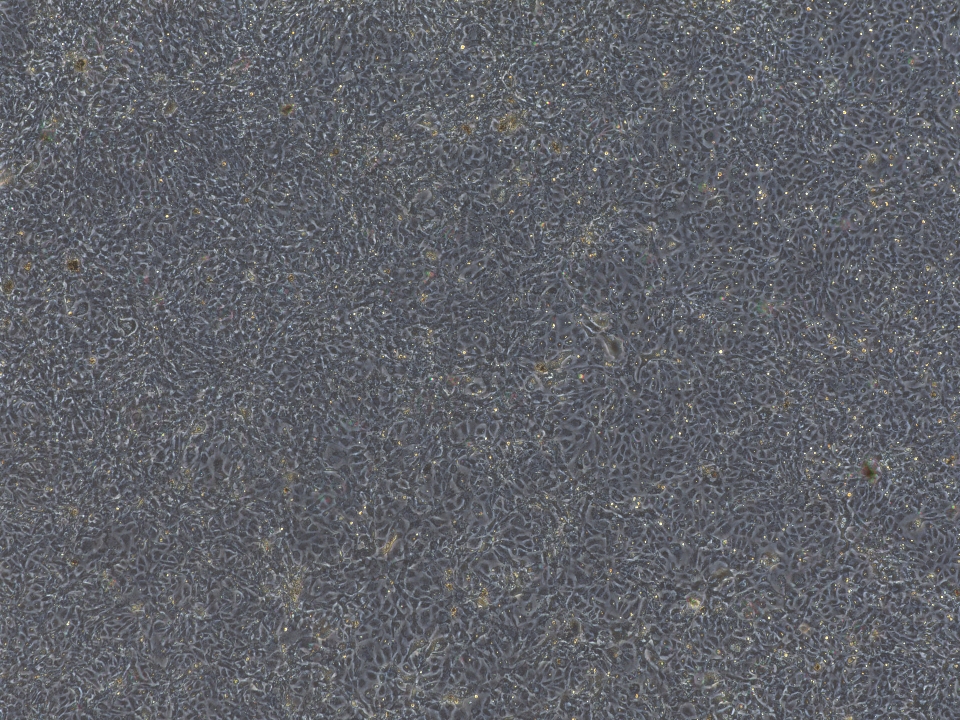

Supplement: S2 File — (ZIP) [file pone.0345921.s008.zip › Raw data for S1-5 Fig/S2 Fig/TrypLE before.jpg]

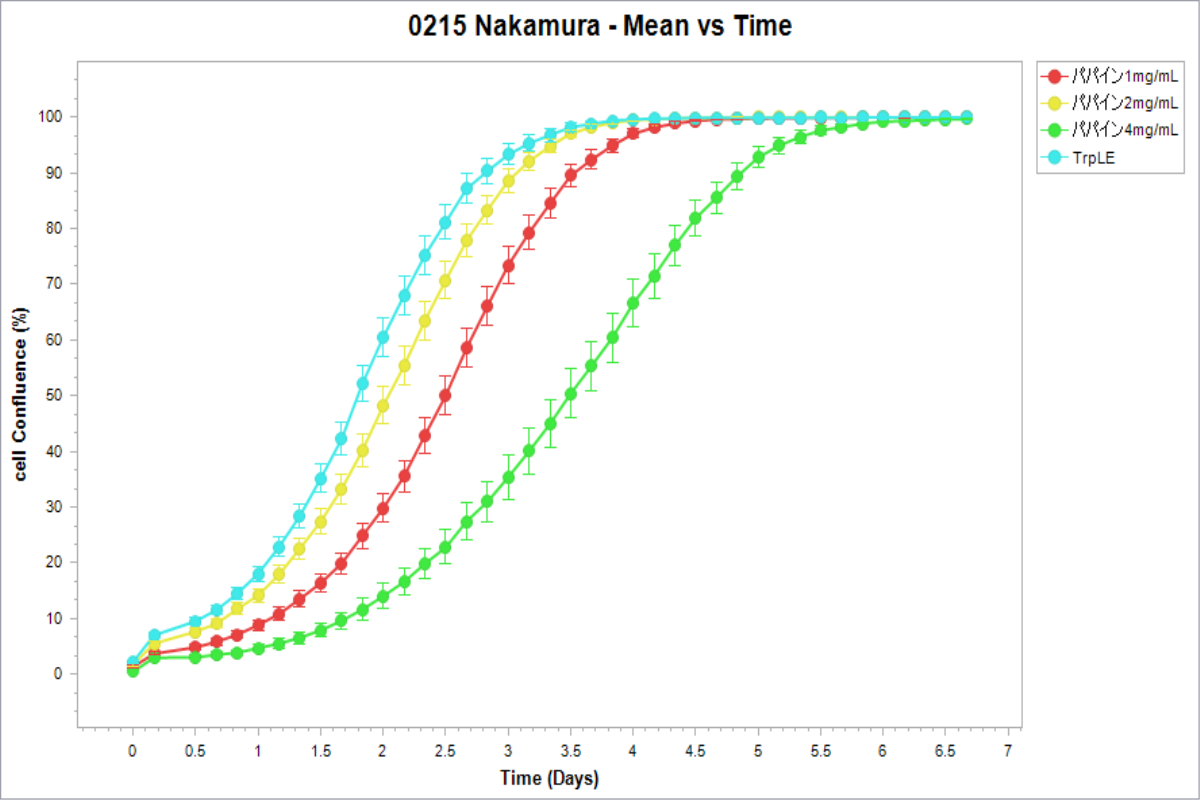

Supplement: S2 File — (ZIP) [file pone.0345921.s008.zip › Raw data for S1-5 Fig/S3 Fig/S3 Fig.png]

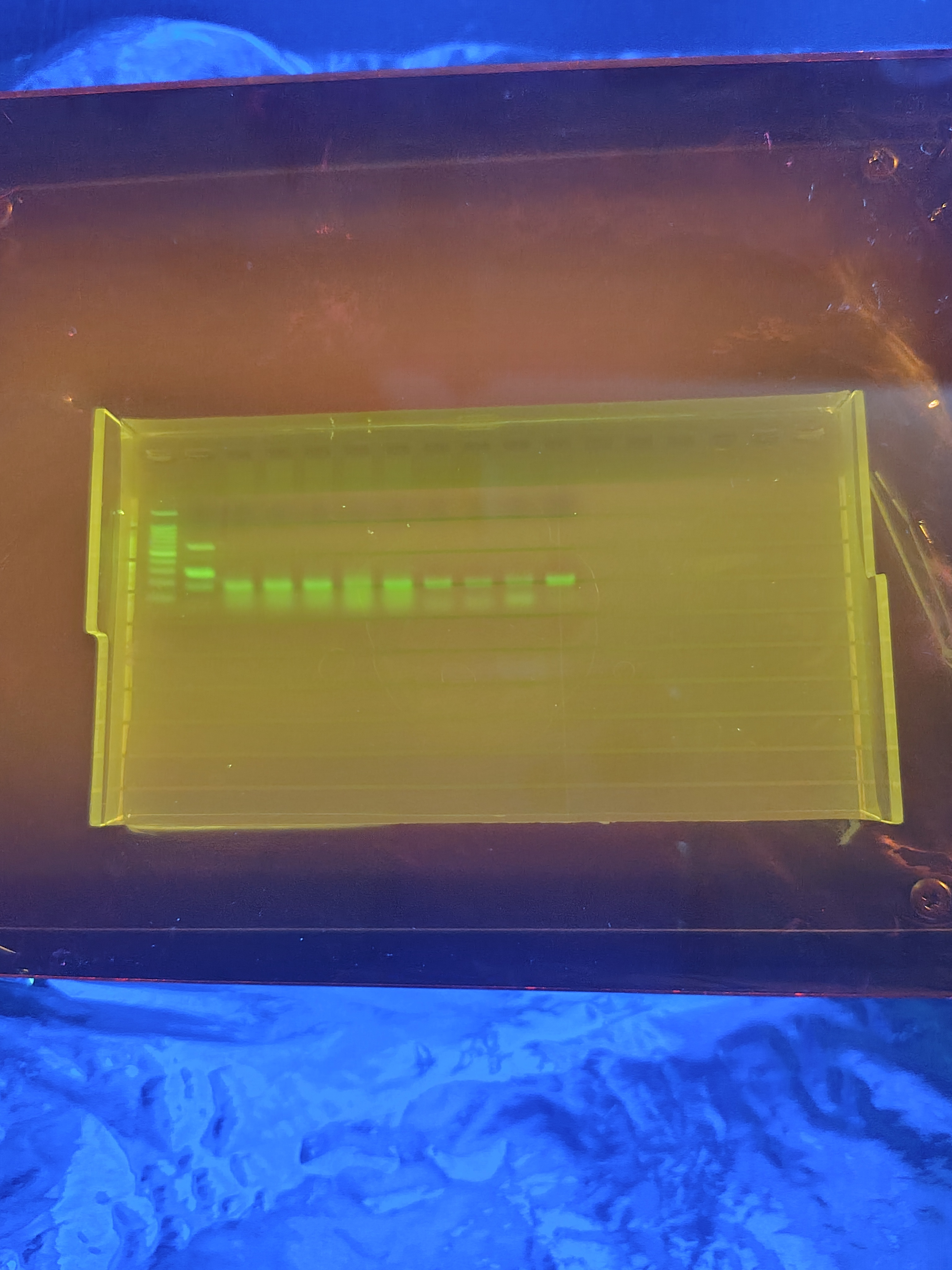

Supplement: S2 File — (ZIP) [file pone.0345921.s008.zip › Raw data for S1-5 Fig/S4 Fig/Validation of sterility_ Mycoplasma PCR test.JPG]
