# Supplementary material for: A food-grade cell dissociation agent via regulatory pre-check framework
Source: PLoS One. 2026 Apr 13;21(4):e0345921. doi: 10.1371/journal.pone.0345921 (PMC13075713; doi:10.1371/journal.pone.0345921)
Supplement: S1 Table — This label demonstrates our framework for avoiding unknown risks in favor of managing known risks. As established in our previous study [20], classifying products as food and providing transparent information is fundamental for reliable safety management in cellular agriculture. (DOCX) [file pone.0345921.s006.docx]

**S1 Table. Proposed food label for iDisper designed to declare its 'manageable risk'.** This label demonstrates our framework for avoiding unknown risks in favor of managing known risks. As established in our previous study [20], classifying products as food and providing transparent information is fundamental for reliable safety management in cellular agriculture.

| Name | Enzyme Preparation (For Food Processing) ^#^ |
| --- | --- |
| Ingredients | Potassium Chloride, Papain (plant-derived), Trisodium Citrate |
| Contents | 100 mL |
| Best Before | 5 months from the manufacturing date ^##^ |
| Storage | Store in a cool, dry place away from direct sunlight. Reconstituted Solution: Store at or below -20°C. |
| Manufacturer & Seller | ”Company’s Name”  “Company's Physical Address” |
| Allergen Information | Contains enzymes derived from papaya. |

^#^ The proposed product name "iDisper" and the associated food label shown here are provisional and intended solely as part of this study. They represent a model case for discussion, and not a finalized commercial specification.

^##^ Although iDisper maintained performance for up to 8 months at −20°C, we conservatively apply a safety factor of 0.7. Accordingly, the recommended guaranteed shelf life is 5 months.
