# Supplementary material for: A food-grade cell dissociation agent via regulatory pre-check framework
Source: PLoS One. 2026 Apr 13;21(4):e0345921. doi: 10.1371/journal.pone.0345921 (PMC13075713; doi:10.1371/journal.pone.0345921)

Cell Count Report

File name

hpc-5

Date

20 Dec., 2022 21:55

Cell count results

Total cell concentration: 1.25 x 10^6 cells/mL

Live cell concentration: 9.72 x 10^5 cells/mL

Dead cell concentration: 2.82 x 10^5 cells/mL

Viability: 77.5 %

Average cell size: 11.6 µm

Total cell number: 262

Live cell number: 203

Dead cell number: 59

Protocol

Protocol name: high sens

Dilution factor: 2

Min. cell size: 3 µm

Max. cell size: 60 µm

Size gating: 3 ~ 60 µm

Noise reduction: 3

Live cell sensitivity: 8

Roundness: 60 %

Declustering level: High

Focusing method: Autofocus

Staining option: With TB

Counting option: Auto exposure(0x045C)

Cell Images (Average intensity: 161)

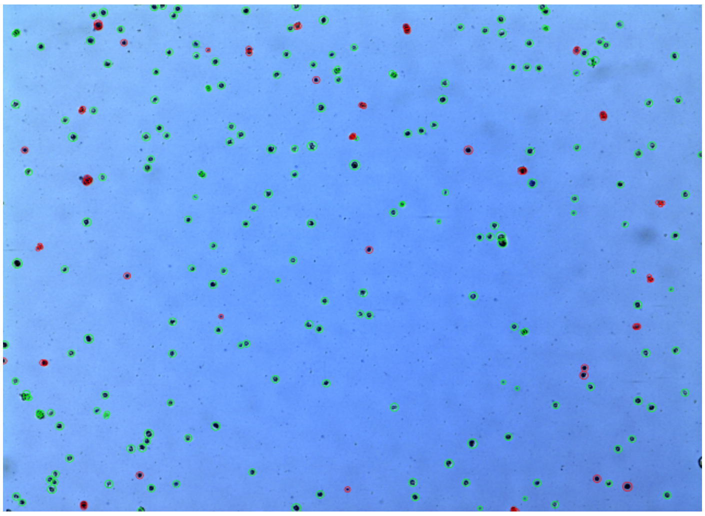

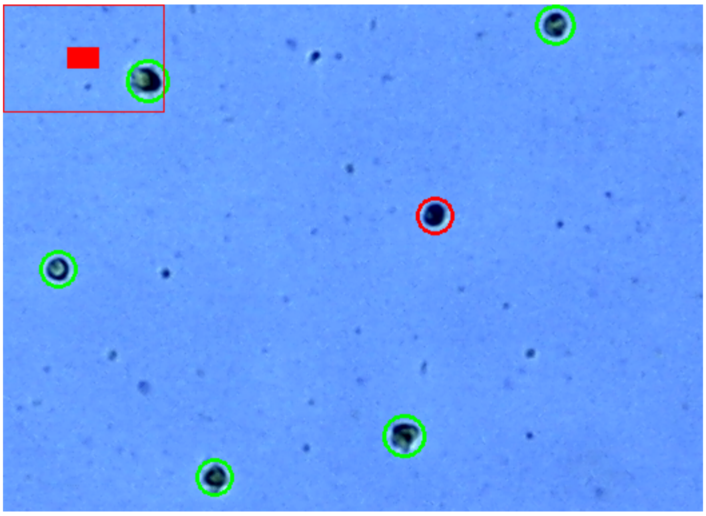

S/N: LUC-06-00807

S/W version: 2.3.3

www.logosbio.com logos biosystems

• Cell size distribution by cell number

Total Cells	Live Cells	Dead Cells

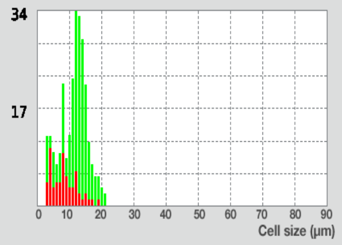

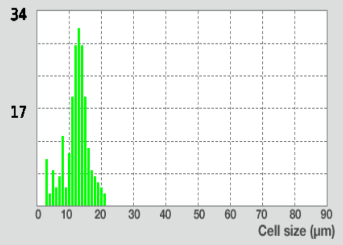

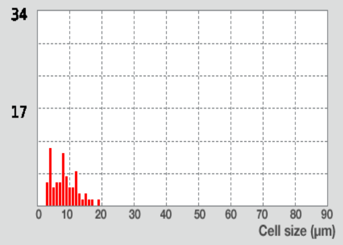

• Cell size distribution by cell concentration

Total Cells	Live Cells	Dead Cells

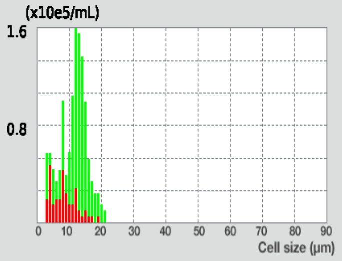

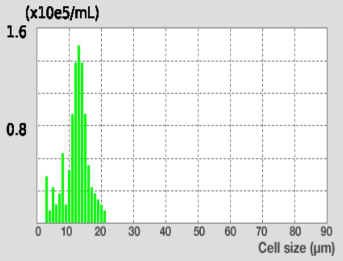

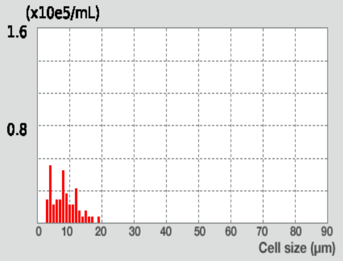

• Cell cluster map

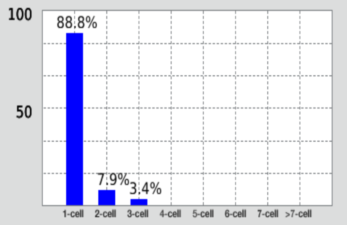

Supplement: S2 File — (ZIP) [file pone.0345921.s008.zip › Raw data for S1-5 Fig/S1 Fig/S1 Fig +Papain+Trisodium Citrate/hpc-5.pdf πü«πé│πâöπüE.pdf]
