# Supplementary material for: A food-grade cell dissociation agent via regulatory pre-check framework
Source: PLoS One. 2026 Apr 13;21(4):e0345921. doi: 10.1371/journal.pone.0345921 (PMC13075713; doi:10.1371/journal.pone.0345921)

Cell Count Report

• File name

cirate10

• Date

20 Dec., 2022 22:56

• Cell count results

Total cell concentration: 2.87 x 10^5 cells/mL

Live cell concentration: 2.15 x 10^5 cells/mL

Dead cell concentration: 7.18 x 10^4 cells/mL

Viability: 75.0 %

Average cell size: 12.7 µm

Total cell number: 60

Live cell number: 45

Dead cell number: 15

• Protocol

Protocol name: high sens

Dilution factor: 2

Min. cell size: 3 µm

Max. cell size: 60 µm

Size gating: 3 ~ 60 µm

Noise reduction: 3

Live cell sensitivity: 8

Roundness: 60 %

Declustering level: High

Focusing method: Autofocus

Staining option: With TB

Counting option: Auto exposure(0x041C)

Cell Images (Average intensity: 154)

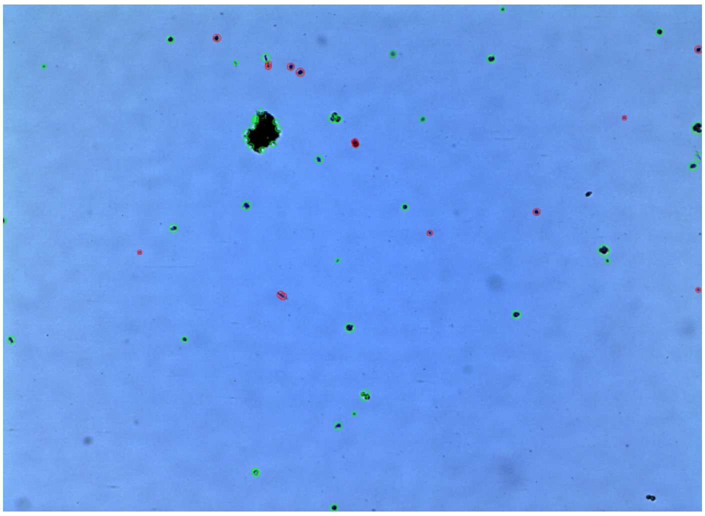

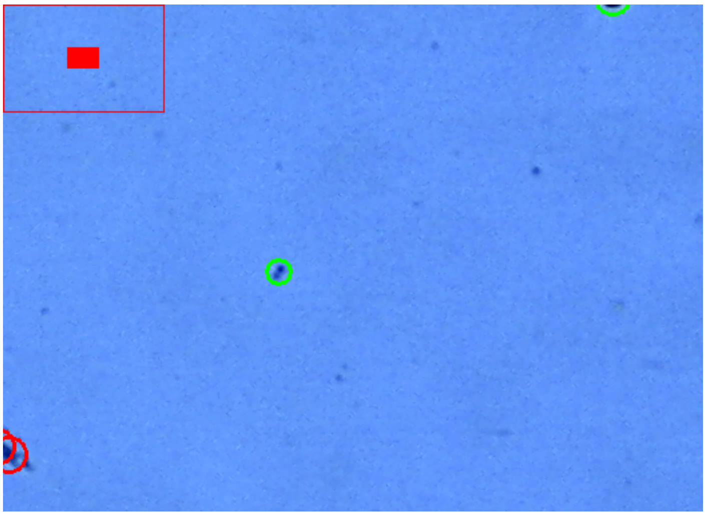

Cell Count Report

• Cell size distribution by cell number

Total Cells	Live Cells	Dead Cells

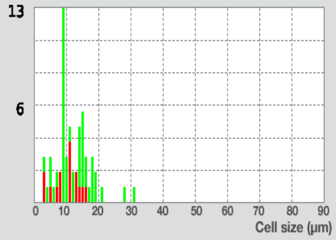

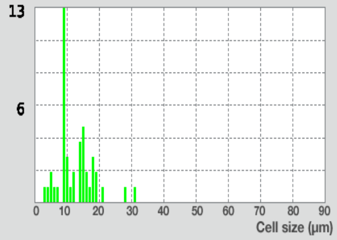

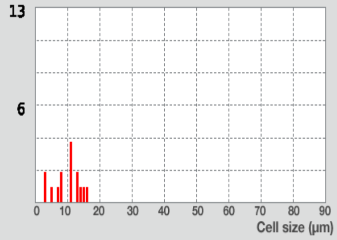

• Cell size distribution by cell concentration

Total Cells	Live Cells	Dead Cells

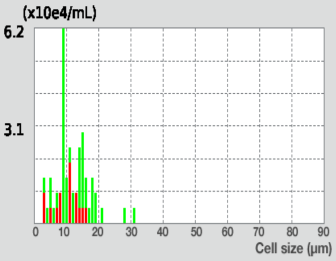

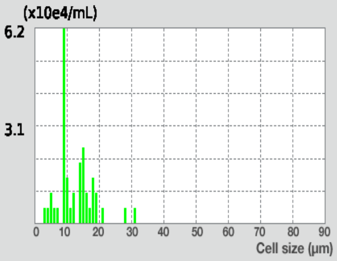

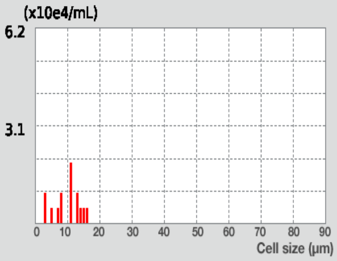

• Cell cluster map

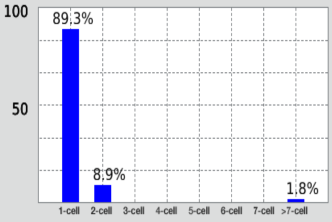

Supplement: S2 File — (ZIP) [file pone.0345921.s008.zip › Raw data for S1-5 Fig/S1 Fig/S1 Fig +Trisodium citrate/cirate10.pdf πü«πé│πâöπüE.pdf]
