# Supplementary material for: A food-grade cell dissociation agent via regulatory pre-check framework
Source: PLoS One. 2026 Apr 13;21(4):e0345921. doi: 10.1371/journal.pone.0345921 (PMC13075713; doi:10.1371/journal.pone.0345921)

# Cell Count Report



## • File name

tryple1

## • Date

20 Dec., 2022 20:58

## • Cell count results

Total cell concentration: 4.69 x $10^5$ cells/mL

Live cell concentration: 3.69 x $10^5$ cells/mL

Dead cell concentration: 1.00 x $10^5$ cells/mL

Viability: 78.6 %

Average cell size: 13.7 µm

Total cell number: 98

Live cell number: 77

Dead cell number: 21

## • Protocol

Protocol name: high sens

Dilution factor: 2

Min. cell size: 3 µm

Max. cell size: 60 µm

Size gating: 3 ~ 60 µm

Noise reduction: 3

Live cell sensitivity: 8

Roundness: 60 %

Declustering level: High

Focusing method: Autofocus

Staining option: With TB

Counting option: Auto exposure(0x041C)

## Cell Images (Average intensity: 152)

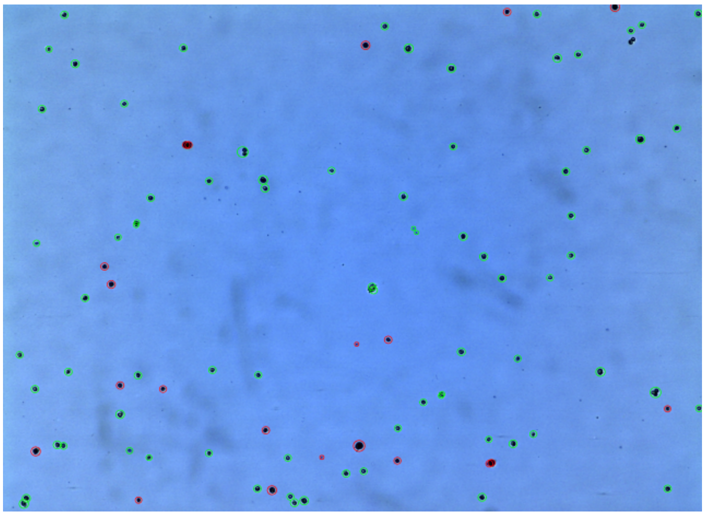

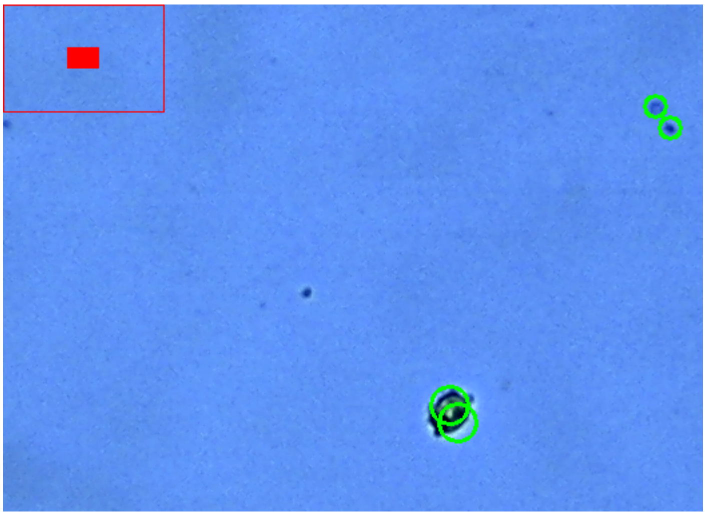

# Cell Count Report



## • Cell size distribution by cell number

| Total Cells | Live Cells | Dead Cells |
|---|---|---|

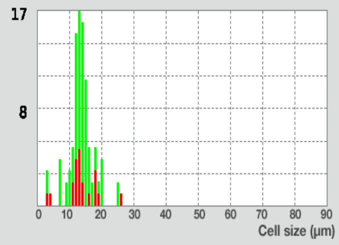


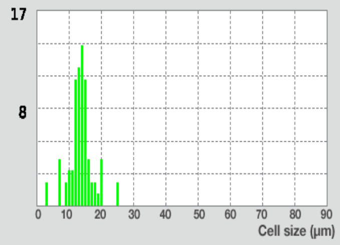


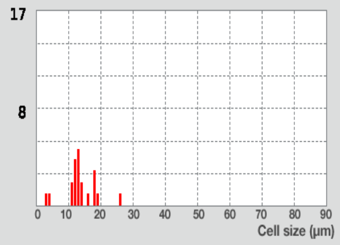


## • Cell size distribution by cell concentration

| Total Cells | Live Cells | Dead Cells |
|---|---|---|

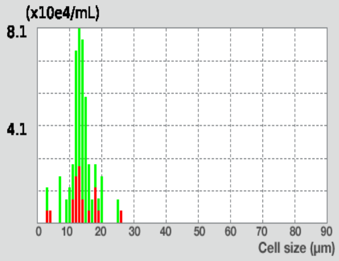


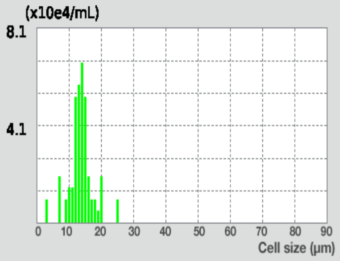


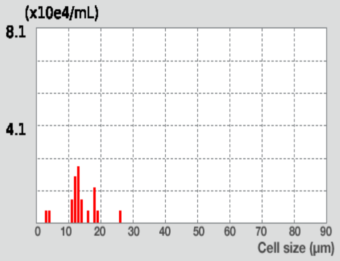


## • Cell cluster map

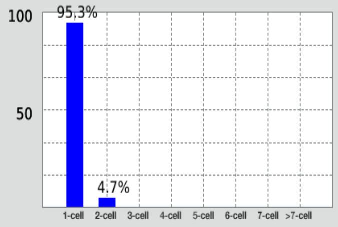

Supplement: S2 File — (ZIP) [file pone.0345921.s008.zip › Raw data for S1-5 Fig/S1 Fig/S1 Fig Commercial Reagent/tryple1.pdf πü«πé│πâöπüE.pdf]
