# Supplementary material for: A food-grade cell dissociation agent via regulatory pre-check framework
Source: PLoS One. 2026 Apr 13;21(4):e0345921. doi: 10.1371/journal.pone.0345921 (PMC13075713; doi:10.1371/journal.pone.0345921)

Cell Count Report

File name

ka13

Date

20 Dec., 2022 23:19

Cell count results

Total cell concentration: 3.11 x 10^5 cells/mL

Live cell concentration: 1.72 x 10^5 cells/mL

Dead cell concentration: 1.39 x 10^5 cells/mL

Viability: 55.4 %

Average cell size: 11.5 µm

Total cell number: 65

Live cell number: 36

Dead cell number: 29

Protocol

Protocol name: high sens

Dilution factor: 2

Min. cell size: 3 µm

Max. cell size: 60 µm

Size gating: 3 ~ 60 µm

Noise reduction: 3

Live cell sensitivity: 8

Roundness: 60 %

Declustering level: High

Focusing method: Autofocus

Staining option: With TB

Counting option: Auto exposure(0x041C)

Cell Images (Average intensity: 155)

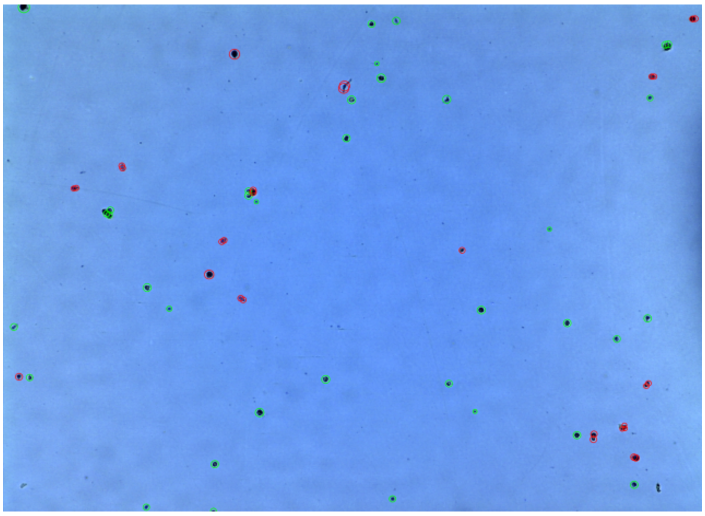

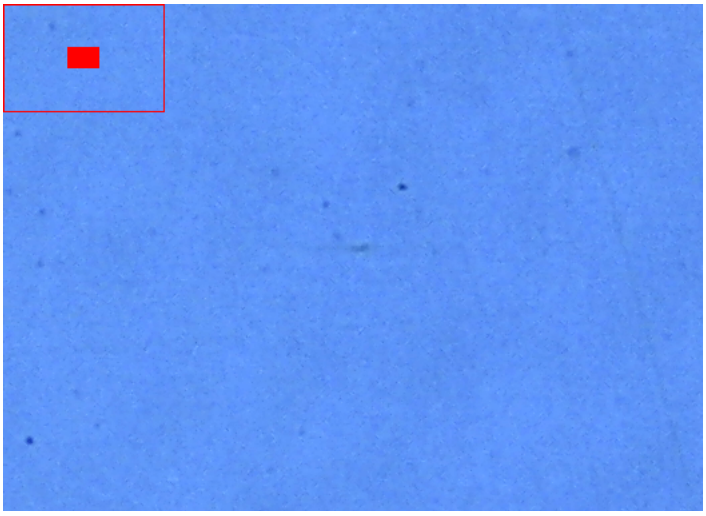

Cell Count Report

• Cell size distribution by cell number

Total Cells	Live Cells	Dead Cells

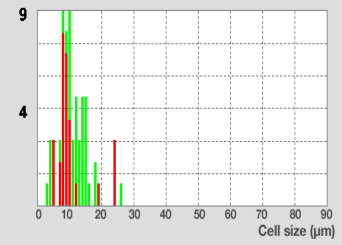

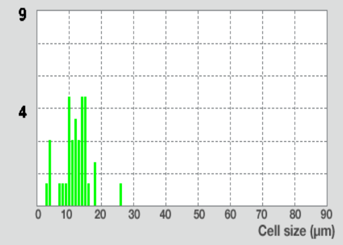

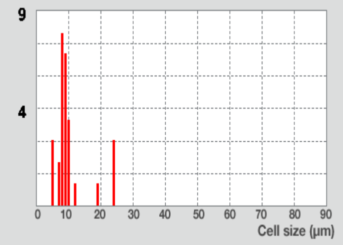

• Cell size distribution by cell concentration

Total Cells	Live Cells	Dead Cells

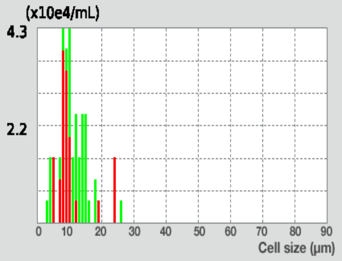

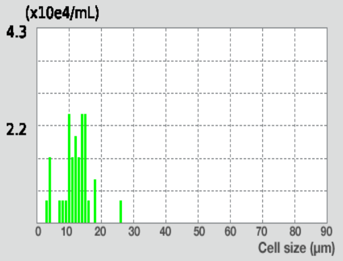

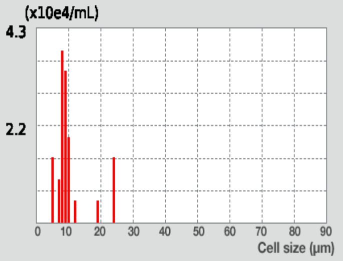

• Cell cluster map

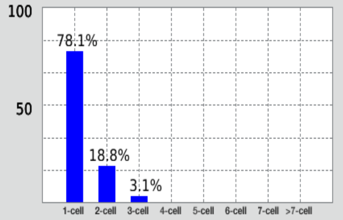

Supplement: S2 File — (ZIP) [file pone.0345921.s008.zip › Raw data for S1-5 Fig/S1 Fig/S1 Fig Negative control/ka13.pdf πü«πé│πâöπüE.pdf]
