# Supplementary material for: A food-grade cell dissociation agent via regulatory pre-check framework
Source: PLoS One. 2026 Apr 13;21(4):e0345921. doi: 10.1371/journal.pone.0345921 (PMC13075713; doi:10.1371/journal.pone.0345921)
Supplement: Attachment — Submitted filename: Response Letter 20260128.docx [file pone.0345921.s010.docx]

**Dear Editor,**

Resubmission of Manuscript ID: PONE-D-25-59887

Title: “A Food-Grade, Animal-Free Cell Dissociation Alternative Product Developed via a Food Regulatory Pre-Check Framework”.

**Response to Editor and Reviewers**

We would like to express our sincere appreciation of the reviewers for their valuable comments and suggestions. We revised the manuscript based on the comments and suggestions provided below. In this response letter, *the reviewers*’ comments are presented in italics, the authors’ responses are written in regular font with a five-character indent, and the revised text is in blue.

**Editor**

We sincerely appreciate the reviewer’s constructive and insightful feedback, which has greatly improved the clarity and quality of our manuscript. We have carefully revised our manuscript in response to these comments. We hope that these revisions adequately address the reviewers’ concerns and that the revised manuscript is now suitable for publication in *PLOS One*. We look forward to hearing from you.

Journal Requirements

*1. Please ensure that your manuscript meets PLOS ONE's style requirements, including those for file naming*

**Response 1**: We appreciate the guidance. We have carefully reviewed the guidelines and revised the manuscript, including file naming conventions and formatting, to fully comply with *PLOS ONE*’s style requirements.

2. Please state what role the funders took in the study.

**Response 2**: We have included the specific role of the funders in the **Cover Letter** (page 2).
**Added text**: This study was supported by the Small Business Innovation Research Program of the Ministry of Agriculture, Forestry and Fisheries of Japan under the “Demonstration of a Production System for Cellular Foods Utilizing CulNet Supernatant” (N020) project. The funders had no role in the study design, data collection and analysis, decision to publish, or preparation of the manuscript.

*3.1. Please provide an amended Funding Statement declaring this commercial affiliation, as well as a statement regarding the Role of Funders in your study.*

**Response 3.1**: We have amended the **Funding Statement** to clarify the commercial affiliation and role of the funder.
**Added text**: The funder provided support in the form of salaries for the authors [HH, MS, MK, AH, HK, and IK], but did not have any additional role in the study design, data collection and analysis, decision to publish, or preparation of the manuscript. The specific roles of these authors are articulated in the ‘author contributions’ section.

*3.2. Please also provide an updated Competing Interests Statement declaring this commercial affiliation along with any other relevant declarations relating to employment, consultancy, patents, products in development, or marketed products, etc.*

**Response 3.2**: We have updated both the **Cover Letter** and **Competing Interests Statement** to explicitly declare commercial affiliation and confirm adherence to journal policies.

**Added text**: This does not alter our adherence to *PLOS ONE* policies on sharing data and materials.

*4. When completing the data availability statement of the submission form, you indicated that you will make your data available on acceptance. We strongly recommend all authors decide on a data sharing plan before acceptance, as the process can be lengthy and hold up publication timelines. Please note that, though access restrictions are acceptable now, your entire data will need to be made freely accessible if your manuscript is accepted for publication.*

**Response 4**: We acknowledge the policy regarding data availability. We confirm that all data underlying the findings described in this manuscript are fully available without restrictions. The minimal data set underlying the results, including the data in Figures 2 and 3, is provided in the **Supporting Information** submitted for this revision.

*5. PLOS requires an ORCID iD for the corresponding author in Editorial Manager on papers submitted after December 6th, 2016. Please ensure that you have an ORCID iD and that it is validated in Editorial Manager. To do this, go to ‘Update my Information’ (in the upper left-hand corner of the main menu), and click on the Fetch/Validate link next to the ORCID field. This will take you to the ORCID site and allow you to create a new iD or authenticate a pre-existing iD in Editorial Manager.*

**Response 5**: The corresponding author (Ikko Kawashima) has a registered **ORCID iD [0009-0003-9612-4007]**. We ensured that this ID was successfully linked and validated using an Editorial Manager System.

*6. We notice that your supplementary table is included in the manuscript file. Please remove them and upload them with the file type 'Supporting Information'. Please ensure that each Supporting Information file has a legend listed in the manuscript after the references list.*

**Response 6**: We appreciate your guidance on the formatting. We have removed the supplementary table from the main manuscript file and uploaded it as a separate file labeled '**Supporting Information**' (S1 Table). Additionally, we have ensured that the legends for all the Supporting Information files are correctly listed at the end of the main manuscript, following the References section.

**Reviewer: 1**

**Comment 1**: *Abstract and Language Issues: Grammatical errors (e.g., "Advancement... require" should be "requires"; "demon-strated" hyphenation artifact) and awkward phrasing diminish the overall clarity. Revise the abstract for clarity and rectify any typographical or formatting mistakes present.*

**Response 1**: We apologize for grammatical errors and awkward phrasing in the original submission. We have thoroughly revised the manuscript, including the abstract, and have had the text proofread by Cactus Communications, Inc. (Tokyo, Japan). Specific corrections, such as "**Advancement... requires**", and “**demonstrated**” have been made.

**Comment 2-1:** *The "Food Regulatory Pre-Check" framework (Fig 1, Table 1) presents an intriguing concept; however, it falls short in terms of thorough validation. Present numerical data that contrasts development timelines and costs with traditional methods, and enhance Table 1 by incorporating global regulatory information beyond Japan, such as the FDA GRAS status for papain. Explain why this represents a "new" framework instead of conventional food additive screening.*

**Response 2-1:** We appreciate the reviewer's suggestion. Although specific financial data and timelines are proprietary and difficult to benchmark owing to the nascency of the industry, we quantitatively validated the framework's efficiency by demonstrating the "Reduction of Search Space." Unlike traditional screening of effectively infinite chemical libraries, our framework restricts the candidate pool to a finite "Positive List" (e.g., 104 enzyme preparations in the WHO JECFA Database; verified by "Enzyme Preparation" in the JECFA Online Database on January 16, 2026;
<https://apps.who.int/food-additives-contaminants-jecfa-database/>) from Day 0. This mathematical constraint to have a significantly higher screening efficiency than conventional methods. We have incorporated this rationale into the Discussion section. Additionally, we updated Table 1 to include regulatory information from Singapore (first approval in 2020), the USA (approval in 2023), and Israel (approval in 2024), which currently lead the global cellular agriculture sector.

**Revised text (Page 8, Line 168)**: The efficiency of this framework is quantitatively supported by the "reduction of search space." Traditional reagent development often requires the screening of vast, effectively infinite libraries of synthetic compounds. In contrast, this framework utilizes the "Positive List" system of food regulations as a beneficial constraint, immediately reducing the candidate pool from an infinite number to a finite, manageable list at the start of development (Day 0). For example, the number of enzyme preparations with established specifications in the WHO JECFA Database [19] is limited to approximately 100 distinct source organisms. This drastic reduction in the screening scope mathematically validates the high efficiency of this framework compared with conventional blind screening.

**Comment 2-2:** *Explain why this represents a "new" framework instead of conventional food additive screening.*

**Response 2-1:** We appreciate the reviewer's comment. **The novelty of this framework is that we reversed the screening order**. Instead of just looking for "what works," we prioritize "what is allowed" to address three practical realities:

- **Global Rules:** Conventional methods often identify high-performing ingredients that are banned in some countries. Our method prevents this by checking global regulations *first*.
- **Cultural Safety:** Food is a culture. As shown by the inclusion of **Kosher status (Table 1)**, we checked religious and cultural suitability early to avoid ethical conflicts.
- **Business Reality:** Many companies find it difficult to stop a project once the budget is approved (Step 3). By setting a mandatory "Pre-Check" (Step 2), we create a clear "Stop/Go" point *before* spending money. Making this common-sense management rule a formal framework is a novel contribution.

**Revised text (Page 9, Line 176)**: This framework also addresses practical business and cultural issues. First, checking regulations early ensures that the product complies with global standards. Second, it ensures "cultural safety" by including religious criteria (e.g., Kosher status in Table 1) from the start. Finally, it aids corporate decision-making. In business, it is often difficult to stop a project once a budget is assigned for testing (Feasibility Test). By requiring a regulatory "Pre-Check," this framework creates a clear decision point to prevent wasted resources and establishes a formal standard for the industry.

**Revised text (Page 9, Line 183)**: Fig 1. "Food Regulatory Pre-Check" upstream development framework. The proposed four-step process begins with (1) Concept Planning, where markets are defined. The core of the framework is (2) Ingredient Screening utilizes the "Food Regulation Pre-Check" (yellow box) as a gatekeeper. This step filters infinite candidates down to a finite compliant list based on global regulations and cultural safety (e.g., Kosher status). Viable candidates then undergo (3) a Feasibility Test, where performance is validated against key metrics. Finally, the lead candidate proceeds to (4) Product Profile Definition, which marks the transition to the downstream development process.

**Comment 3:** *The methods presented are lacking in detail: The sourcing of cells from duck liver and chicken muscle does not include necessary ethical approvals, provenance information, or controls for variability. The preparation of iDisper lacks specific details regarding pH levels, mixing conditions, and does not provide validation for sterility beyond the filtration process. It is essential to ensure that statistical methods are supported by the deposition of raw data (ChemRxiv DOI provided, but please verify accessibility). Incorporate comprehensive protocols to ensure reproducibility.*

**Response 3:** We appreciate the reviewer’s guidance on improving the transparency and reproducibility of our methods. We have addressed each point as follows:

**Ethical Information**: We added a specific statement regarding ethical compliance to the manuscript.
Revised text (Page 16, Line 295): **Compliance with Ethical Standards**
In this study, we used duck eggs embryonated before hatching. According to Japanese institutional guidelines and the SCAW Category A classification, these embryos are not defined as experimental animals; therefore, formal approval from the Institutional Animal Care and Use Committee was not required. All procedures were performed in accordance with the principles of animal welfare to minimize potential distress.

**Provenance Information of duck liver and chicken muscle cells**: We have expanded the "Materials and Methods" section to include detailed preparation conditions and quality control specifications to ensure reproducibility.
Revised text (Page 4, Line 77): **Cell Source, Cell Culture and Evaluation Method**
Primary cells were isolated from the duck liver and chicken muscle. Fertilized duck eggs were purchased from Shiina Hatchery (Japan), and fertilized chicken eggs were obtained from Yamagishi (Wakayama, Japan). Both types of eggs were incubated in a P-008 incubator (Showa Furanki, Saitama, Japan) maintained at 37 °C and 60% humidity. The incubation periods were set to 14 and 12 days for the duck and chicken eggs, respectively, to ensure appropriate embryonic development. To ensure sterility, the egg surface was sterilized with ethanol, followed by immersion in sodium hypochlorite solution (Kitchen Haiter; Kao, Japan) for 30 min. Embryos were aseptically extracted in a laminar flow hood, and liver tissues were dissected. The tissues were mechanically dissociated in I-MEM without enzymes using a gentleMACS™ Dissociator (Miltenyi Biotec, Germany). The cell suspension was centrifuged, and the pellet derived from the two embryos was pooled and seeded into a single T175 flask to minimize individual variability.

**iDisper Preparation and Sterility Validation**: We expanded the "Materials and Methods" section to include detailed preparation conditions and quality control specifications to ensure reproducibility.

Revised text (Page 4, Line 62): The iDisper was prepared by mixing food-grade papain (W-40; Amano Enzyme, Aichi, japan), trisodium citrate (Marugo Corporation, Saitama, Japan), and potassium chloride (Kanto Chemical, Tokyo, Japan) in purified water at final concentrations of 2, 0.37, and 28.72 mg/mL, respectively. The reagents were weighed using a precision electronic balance (FX-5000i; A&D, Tokyo, Japan) and added sequentially to water. To ensure complete dissolution, the mixture was stirred at 550 rpm using a magnetic stirrer for 1 h at room temperature (25°C). The solution was sterilized using a Nalgene™ Rapid-Flow™ Sterile Disposable Filter Unit (0.2 µm pore size; 431433; Thermo Scientific, MA, USA). iDisper was stored at −20 °C until use. Before use, the solution was thawed and heated to 37 °C in a water bath. The physicochemical properties were verified using a pH meter (FP20; Mettler Toledo, Switzerland) and an osmometer (Osmostat OM-6040; ARKRAY, Tokyo, Japan). The pH was measured to ensure it fell within the range of 5.5–6.0 without adjustment, and osmolality was adjusted to 295±5 mOsm/kg. To validate sterility, the filtered solution was plated on Soybean-Casein Digest (SCD) agar and incubated at 35°C. Colony-forming units (CFU) were quantified using an automatic colony counter (Scan500; Interscience, Saint Nom la Bretêche, France). Internal quality control records confirmed 0 CFU across independent manufacturing lots. iDisper was stored at −20 °C until use.

**Raw Data Accessibility:** Regarding the availability of data, we have uploaded the complete raw dataset underlying our statistical analyses as Supporting Information in this resubmission, rather than relying on the external ChemRxiv repository.

**Comment 4:** *The interpretation of results appears exaggerated: Assertions of "comparable" performance to TrypLE Express (Figs 2-3) overlook significant differences in incubation duration, cell types, and metrics for passaging success. Figure 2d pie charts illustrate significant failure rates (approximately 30-50%) that cannot be accounted for by contamination or reagent-related problems. Examine the constraints of testing exclusively on birds and analyze the duration of papain allergens in a quantitative manner.*

**Response 4**: Thank you for your comment. We have revised the manuscript to explicitly attribute the failure rates to raw material variability and provide a quantitative analysis of allergen stability based on data and literature.

**Clarification of Figure 2d (Failure Rates)**: We revised the manuscript to explicitly attribute the failure rates to the current limitations of iDisper during the primary isolation phase. Clarification of Figure 2d (Failure Rates): We clarified that the failure rates shown in Figure 2d, particularly the 4.1% failure rate at passage 0 (P0), highlighted a specific challenge. Although iDisper is highly effective for cell expansion (passage 1 onwards), the P0 data suggests that iDisper has not yet been fully optimized to overcome the inherent variability of primary tissue isolation. We have revised the text to acknowledge that this instability could potentially reduce overall production efficiency, identifying it as a priority for future product optimization.

Revised text (Page 12, Line 213): Furthermore, iDisper consistently supported robust expansion through serial passaging, simulating a cell-based food production line (Fig 2d-2e). However, the 4.1% failure rate at passage 0 indicates a limitation of iDisper in overcoming the inherent variability in primary avian cells. This instability at the initial isolation stage acts as a bottleneck, potentially reducing overall production efficiency. Therefore, optimizing the iDisper for robust primary isolation while being effective for expansion remains a critical challenge.

**Quantitative Analysis of Papain Allergens**: We concluded that the allergen remaineds fully active and did not naturally degrade throughout the manufacturing process. This structural stability is quantitatively supported by our internal data (Fig 3b), which shows robustness for 8 months at -20°C, and by Vicente et al. (1995) [25], which confirms a half-life of approximately 10 days at room temperature. These data demonstrated that allergens persisted as risk factors throughout the operational window.

Revised text (Page 15, Line 270): In terms of temporal stability, the allergen remained structurally robust throughout the process. Our data (Fig 3b) showed stability for 8 months at -20°C, and Vicente et al. [25] reported that papain retains its stability with a half-life of 10 days in aqueous media. This quantitative evidence indicates that the allergen does not degrade naturally during cell dissociation, necessitating active removal strategies.

[25] Vicente, L. C., Aires-Barros, R., & Empis, J. M. Stability and proteolytic activity of papain in reverse micellar and aqueous media: a kinetic and spectroscopic study. *J. Chem. Technol. Biotechnol.,* 1994, 60.3, 291-297. https://doi: 10.1002/jctb.280600310.

**Comment 5:** *Concerns regarding conflict of interest and commercial bias arise from the involvement of all authors from IntegriCulture Inc. (the product developer), particularly in relation to provisional labeling (S1 Table). Offer unbiased validation data or external testing; reduce promotional language (e.g., "Everyone Can Make Cell-Based Food").*

**Response 5**: We appreciate the reviewer’s feedback regarding the potential commercial bias. To address this issue and ensure an objective scientific tone, we have made the following revisions:

**Generalization of S1 Table**: To clarify that S1 Table is intended as a general regulatory framework model rather than a specific product advertisement, we have removed the specific company details. The "Manufacturer & Seller" section has been changed from "IntegriCulture Inc." and its specific address to generic placeholders: "Company’s Name" and "Company's Physical Address."

**Removal of Promotional Language**: We removed promotional phrases from the manuscript. Specifically, we deleted the vision statement "embody our vision of Everyone Can Make Cell-Based Food" from the Discussion section to maintain a neutral perspective.

Reviewer: 2

**Comment 6:** *You state that iDisper could maintain passaging of cells with hepatic stellate identity and stable marker expression, however your results S2 Appendix – S1 indicate a loss of ALB and HNF4A in P2 and P6 cells (both hepatocyte maturation markers). Can you please clarify why you conclude that iDisper maintains cells with hepatic stellate identity i.e. what markers are you looking at to make that conclusion?*

**Response 6**: We agree with the reviewer’s comments. The data demonstrated that, while hepatocyte markers decreased, the expression of mesenchymal markers (COL3A1 and DCN) increased. Therefore, we redefined the cells as presenting a general mesenchymal phenotype rather than a specific hepatic stellate identity.

**Revised text (Page 12, Line 195)**: The iDisper performance was evaluated using an industrially relevant cell population. We used primary duck liver-derived cells, which are used as raw materials for cell-based food. Under standard culture conditions, these cells proliferated as adherent cells and were predominantly mesenchymal cells (S1 Appendix, S1 Fig).

**Revised text (Page 13, Line 219)**: Cells propagated with iDisper remained phenotypically stable, maintaining a mesenchymal phenotype capable of sustained proliferation despite the loss of specific hepatocyte markers (Fig 2e; S1 Fig).

**Comment 7:** *You examined microbial contamination in passaged cells, but did you confirm that cell cultures were mycoplasma free?*

**Response 7**: We appreciate the important question regarding quality control. We confirmed that cells passaged with iDisper were negative for Mycoplasma. Electrophoresis results, showing the absence of mycoplasma bands, are presented in the new S5 Fig.

**Revised text (Page 7, Line 137)**: **Mycoplasma Detection**
To ensure sterility, mycoplasma contamination was assessed in duck liver-derived cells (passages 2 and 5) propagated using the iDisper. Genomic DNA was extracted using the Dneasy® Blood & Tissue Kit (69504; Qiagen, Germany) according to the manufacturer's instructions. PCR amplification was performed using the e-Myco™ plus Mycoplasma PCR Detection Kit (25237; iNtRON Biotechnology, Korea). The amplification products were visualized using gel electrophoresis to confirm the absence of mycoplasma bands (S5 Fig).

**Revised text (Page 13, Line 221)**: Mycoplasma testing confirmed that the cells passaged with different lots of iDisper (P2 and P5) were negative for contamination (S5 Fig).

**Revised text (Page 23, Line 418)**: **S5 Fig. Validation of sterility: Mycoplasma PCR test.**
Duck liver cells passaged with iDisper were tested using the e-Myco™ plus Kit across three independent lots (Lots A, B, and C). Lanes: Marker: DNA ladder; Positive Control (kit): Positive control showing specific target bands (including 270 bp); Duck liver-derived cell (P2, Lot A): Cells at Passage 2 (*n*=3); Duck liver-derived cell (P2, Lot B): Cells at Passage 2 (*n*=3); Duck liver-derived cell (P5, Lot C): Cells at Passage 5 (*n*=2); Water: Negative control. The presence of internal control bands in all sample lanes confirms successful PCR amplification, while the absence of the specific 270 bp band confirms that all cell cultures were free of mycoplasma contamination.

**Note on Figure Numbering**: To insert these new data into the logical order of the results, we designated the Mycoplasma data as S5 Fig. Consequently, the original S5 Fig. (long-term stability) was renumbered to S6 Fig.

**Comment 8:** *There is no discussion of Sup Fig 3 and 4 in the main manuscript – please include discussion especially since you conclude that papain with trisodium citrate is better than papain alone. You have stated that papain alone could not achieve dissociation to the levels of papain with trisodium citrate (line 133), however you currently have not presented/discussed the results to support that claim (or please move your S2 Appendix discussions into the main body of the manuscript). I see that Sup Fig 3 indicates dissociation of duck liver cells with papain alone at various concentrations, and it appears that papain alone did achieve similar dissociation results compared to the TryplE Express reagent. Hence I’m not convinced that papain + trisodium citrate is required over papain alone.*

**Response 8**: Thank you for the suggestion. As requested, we have moved the discussion of the optimization process (S3 and S4 Figs) from the Supporting Information to the main manuscript. Although our results indicated that papain alone was sufficient for dissociation, we intentionally designed iDisper to combine papain with trisodium citrate. This was done to replicate the reliable "dual-action" approach of the industry standard, Trypsin-EDTA. Using both an enzyme and a chelating agent, we ensured that our product worked in the same way to detach cells effectively. We have revised the text to explain this rationale explicitly.

**Revised text (Page 12, Line 200)**: Development of iDisper began with trisodium citrate as a gentle baseline chelating agent, considering the delicacy of primary cells.[19] However, when tested on primary duck liver-derived cells, citrate alone failed to achieve complete detachment, as shown by microscopy and low cell counts (Fig 2a-2c). Particle size analysis indicated the presence of large aggregates with citrate alone (S2 Fig). . To improve efficacy, we investigated the addition of food-grade protease papain. A comparative evaluation demonstrated that the combination of trisodium citrate and papain significantly improved cell dissociation. A papain concentration of 2 mg/mL yielded a dissociated cell number and a single-cell particle size distribution comparable to the commercial reagent TrypLE™ Express (S3 Fig). In contrast, 1 mg/mL was insufficient for effective dissociation. Although a concentration of 4 mg/mL also induced detachment, it caused a significant delay in subsequent cell proliferation compared to 2 mg/mL (S4 Fig). Although papain alone was effective, the iDisper formulation was finalized by combining papain with trisodium citrate. This design was chosen to mimic the dual mechanism of standard trypsin-EDTA reagents, utilizing proteolysis (2 mg/mL papain) and chelation (citrate) to target distinct cell adhesion factors.

**Comment 9:** *Fig 3 – no indication of A, B or C on the figure. Also can you indicate statistical significance on the figures.*

**Response 9**: We appreciate the reviewer’s suggestion to improve clarity of the figures. We have revised Figure 3 to explicitly label the panels as (a), (b), and (c). Furthermore, we have added statistical significance markers to the graphs. As detailed in the revised Materials and Methods and the Figure 3 legend, we performed statistical analyses separately for total and live cell counts using Student's t-test followed by Bonferroni correction. Significant differences compared to the reagent group are indicated by asterisks (* *p* < 0.05, ** *p* < 0.01).

**Revised text (Page 15, Line 249)**: **Fig 3. Versatility and stability of iDisper across different cell types.** Performance of freshly prepared iDisper ("Fresh"), iDisper stored for 8 months at −20 ℃ ("8 month"), and a commercial control ("Reagent") was compared on duck liver-derived cells (a), chicken muscle-derived cells (b), and the DF-1 cell line (c). Blue and black bars represent total and live cell numbers, respectively. Data are presented as mean ± SD (n=3). Statistical analyses were performed separately for total and live cell counts using Student's t-test followed by Bonferroni correction. * *p* < 0.05, ** *p* < 0.01 compared to the Reagent group.

**Comment 10:** *Methods section of the main manuscript could be expanded to include S1 Appendix (do not understand why this methods has been moved to the supplementary section.*

**Response 10**: Thank you for the suggestion. We agree that including the complete methodology in the main text improved the readability and reproducibility of the study. Accordingly, as requested, we moved the detailed methods regarding the iDisper optimization process (previously located in S1 Appendix) to the "Materials and Methods" section of the main manuscript.
Page 7, Line 126: S1. RNA Extraction and Sequencing
Page 8, Line 134: S2. Bioinformatic Analysis
Page 7, Line 114: S3. Cell Detachment Assay
Page 6, Line 99: S4. Cell Expansion

**Note on S1 Appendix Restructurin**g: To improve the readability of the manuscript as requested, the methodological details of the optimization process were moved to the main text. Consequently, S1 Appendix has been restructured and renamed "Supplementary Results" to exclusively present the supplementary data and discussion regarding cell characterization and production success rates.

**Comment 11:** *When washing the dissociated cells with PBS, is there calcium and magnesium included in the PBS?*

**Response 11**: We appreciate the reviewer’s comment regarding the buffer composition. PBS without calcium or magnesium was used to ensure optimal dissociation (particularly for the chelating action of citrate). We have updated the manuscript to explicitly describe the details of the product.

**Revised text (Page 5, Line 98)**: Cell dissociation efficacy was evaluated using 12-well plates. After removing the culture medium from each well and washing once with PBS (D-PBS(-) without Ca and Mg; 048-29805; FUJIFILM Wako, Tokyo, Japan), 1 mL of either pre-warmed iDisper or a control reagent (TrypLE™ Express; 12605010; Thermo Fisher Scientific, MA, USA) was added. The plates were incubated for 5 min, after which the cells were detached by pipetting.

**Comment 12:** *Details in supplemental methods do not match what is written in main manuscript – for example, in S1 Appendix-S3 Cell detachment assay, you state that the Cell Counter model R1 (Olympus) was used to measure total cell number and viability, but in the main manuscript you state that the NucleoCounter NC-202 was used. Please clarify if both equipment were used.*

**Response 12**: We appreciate the reviewer’s comments. Both instruments were used for specific purposes.

NucleoCounter (NC-202) was used for most of the experiments (including Figures 2 and 3) because it provides high reliability in distinguishing live/dead cells using fluorescent dyes (acridine orange and DAPI), which is superior to image-based analysis for our main dataset.

In contrast, Cell Counter model R1 (Olympus) was specifically used for the optimization experiments, as shown in S2 and S3 Figs. This is because during the initial optimization phase, we needed to analyze the particle size distribution to evaluate the presence of cell aggregates caused by insufficient detachment, a function provided by R1.

We have revised the "Materials and Methods" section to explicitly describe the specific use of both instruments.

**Revised text (Page 5, Line 102)**: The resulting cell suspension was collected, and the cell number and viability were measured using an automated cell counter (NucleoCounter® NC-202™; ChemoMetec, Denmark) with Via2-Cassette (941-0024; ChemoMetec, Denmark) containing acridine orange and 4′,6-diamidino-2-phenylindole (DAPI) fluorescent dyes, according to the manufacturer’s instructions.

**Revised text (Page 6, Line 115)**: The live cell number and particle size distribution were determined using the Cell Counter model R1 (Olympus, Tokyo, Japan) in S2, S3 Fig.

**Comment 13:** *Proof-read the manuscript before submission, especially punctuation.*

**Response 13**: Thank you for your comment. We have thoroughly reviewed the manuscript and corrected punctuation errors. The revised manuscript has been proofread by Cactus Communications Inc. (Tokyo, Japan), a professional English editing service, to ensure grammatical accuracy and clarity.

**Comment 14:** *Table 1 format can be improved.*

**Response 14**: Thank you for the suggestion. To enhance readability, we reformatted Table 1 into a landscape orientation and optimized the font size. As mentioned in Response 2, we added regulatory status columns for key international regions—Singapore (approval in 2020), the USA (approval in 2023), and Israel (approval in 2024)—to provide a more comprehensive global perspective.

Thank you for allowing us to strengthen our manuscript with your valuable comments and queries. We hope that these revisions are sufficient to make our manuscript suitable for publication in *PLOS One*, and look forward to hearing from you.

Yours sincerely,

Ikko Kawashima, PhD,

CTO
IntegriCulture Inc.
Email: [ikko@integriculture.com](mailto:ikko@integriculture.com)
Phone: +81 50 5470 9432
