# Supplementary material for: A food-grade cell dissociation agent via regulatory pre-check framework
Source: PLoS One. 2026 Apr 13;21(4):e0345921. doi: 10.1371/journal.pone.0345921 (PMC13075713; doi:10.1371/journal.pone.0345921)
Supplement: Attachment — Submitted filename: Response Letter2.docx [file pone.0345921.s011.docx]

**Dear Editor,**

Resubmission of Manuscript ID: PONE-D-25-59887R1

Title: “Food-grade cell dissociation agent via regulatory pre-check framework”.

**Response to Editor and Reviewers**

We would like to express our sincere appreciation of the reviewers for their valuable comments and suggestions. We revised the manuscript based on the comments and suggestions provided below. In this response letter, *the reviewers*’ comments are presented in italics, the authors’ responses are written in regular font with a five-character indent, and the revised text is in blue.


**Comment 1**: *Abstract and Title: Refine the title for conciseness (e.g., "Food-Grade Cell Dissociation Agent via Regulatory Pre-Check Framework"). Expand abstract to explicitly state key metrics (e.g., 2 mg/mL papain efficacy, 8-month stability).*

**Response 1:** Thank you for your comment. We have revised the title to be more concise. Furthermore, regarding the Abstract, we have explicitly stated the key metrics, including the 2 mg/mL papain efficacy and the 8-month stability, to clarify our findings.

**Revised text (Title)**: Food-grade cell dissociation agent via regulatory pre-check framework

**Revised text (Abstract)**: Advancements in cell-based foods require materials that meet the highest food safety standards. Conventional cell dissociation agents, such as animal-derived trypsin, pose significant risks including, viral contamination and allergenicity, which hinder their use in food production. To overcome this challenge, we propose a food regulatory pre-check framework, which prioritizes regulatory compliance before functional testing. Following this framework, we developed iDisper, a novel food-grade animal-free cell dissociation reagent composed of approved food additives (papain and trisodium citrate). Specifically, an optimized formulation containing 2 mg/mL papain demonstrated a dissociation efficacy and viability comparable to those of commercial agents in primary avian cells. Furthermore, iDisper demonstrated 8-month stability at −20 °C. This study validated the proposed framework as an effective strategy for developing safe and practical reagents for the cell-based food industry.

**Comment 2:** *Introduction: Add 1-2 sentences citing recent cell-based food safety reviews (e.g., refs 3-4) to strengthen context on upstream challenges.*

**Response 2:** Thank you for your comment. We have revised the manuscript to emphasize the context on upstream challenges by adding two sentences citing recent cell-based food safety reviews.

**Revised text (Page 2, Line 27)**: For commercially viable cell-based products, the entire production process must follow the same rigorous safety standards as conventional foods [1-3] In the coming decade, compliance with established food safety management systems, such as HACCP and FSSC 22000, will likely be required.[4] These standards apply not only to final products but also to all raw materials including cell-based food. Therefore, a comprehensive reassessment of manufacturing processes is essential. Recent reports on cell-based product safety emphasize that strictly controlling and characterizing all production inputs —including the composition of cell manipulation reagents— is a critical challenge for regulatory compliance [5, 6]. This necessitates a fundamental shift in development philosophy from the earliest stages of product design; however, such a comprehensive approach has yet to be widely adopted in practice.

[5] World Health Organization (WHO); Food and Agriculture Organization of the United Nations (FAO).; FOOD SAFETY ASPECTS OF CELL-BASED FOOD, **2023**, https://doi.org/10.4060/cc4855en
[6] Kimberly J. O., Jeremiah J., Isha D., Vincent S., Dwayne H., Jo A. S; Food safety considerations and research priorities for the cultured meat and seafood industry. *Compr. Rev. Food Sci. Food Saf.*, **2021**, 6(20), 5421-5448. https://doi.org/10.1111/1541-4337.12853

**Comment 3:** *Methods: Specify exact iDisper osmolality adjustment protocol beyond "adjusted to 295±5 mOsm/kg" and include centrifuge RPM for all steps (e.g., 300 g noted inconsistently). Clarify embryo incubation humidity control details.*

**Response 3-1:** Thank you for pointing this out. We apologize for the inaccurate description in the original manuscript. Upon reviewing our experimental records, we confirmed that both the pH and the osmolality of the iDisper solution naturally fell within the target ranges (5.5–6.0 and 295±5 mOsm/kg, respectively) upon initial preparation. Therefore, no further adjustment protocols were actually performed.

**Revised text (Page 4, Line 75)**: The pH and osmolality were measured to ensure they fell within the ranges of 5.5–6.0 and 295±5 mOsm/kg, respectively, without requiring any further adjustment.

**Response 3-2:** Thank you for pointing this out. We added the all centrifuge condition in this study.
**Revised text (Page 5, Line 88)**: Embryos were aseptically extracted in a laminar flow hood, and liver tissues were dissected. The tissues were mechanically dissociated in I-MEM without enzymes using a gentleMACS™ Dissociator (Miltenyi Biotec, Germany). The cell suspension was centrifuged at 300 × g for 4 min at room temperature, and the pellet derived from the two embryos was pooled and seeded into a single T175 flask to minimize individual variability.
**Revised text (Page 5, Line 94)**: The cells were maintained and cultured under standard conditions (37℃, 5% CO2) in a basal medium, I-MEM, [18] supplemented with 10% Yolk Lipoprotein (YLP), an egg-derived serum substitute. [19] Before cell seeding, the culture vessels were coated with the gelatin-based coating agent iCoater, as described in our previous study. [20] For each passage in the cell-expansion regimen, cells were initially seeded at 25% confluency (5×10^6^ cells/T175 flask) based on these counts and cultured until an approximate 4-fold expansion reached approximately 100% confluency (20×10^6^ cells/T175 flask), at which point they were detached using iDisper. The detached cells were then recovered by centrifugation at 300 × g for 5 min at room temperature. For specific experiments to determine cell doubling times, cells were cultured and analyzed as they reached approximately 70% confluency.
**Revised text (Page 6, Line 103)**: Cell dissociation efficacy was evaluated using 12-well plates. After removing the culture medium from each well and washing once with PBS (D-PBS(-) without Ca and Mg; 048-29805; FUJIFILM Wako, Tokyo, Japan), 1 mL of either pre-warmed iDisper or a control reagent (TrypLE™ Express; 12605010; Thermo Fisher Scientific, MA, USA) was added. The plates were incubated for 5 min, after which the cells were detached by pipetting. The resulting cell suspension was collected and centrifuged at 300 × g for 5 min at room temperature, and the cell number and viability were measured using an automated cell counter (NucleoCounter® NC-202™; ChemoMetec, Denmark) with Via2-Cassette (941-0024; ChemoMetec, Denmark) containing acridine orange and 4′,6-diamidino-2-phenylindole (DAPI) fluorescent dyes, according to the manufacturer’s instructions.
**Revised text (Page 6, Line 113)**: Primary duck-liver-derived cells were seeded in 6-well plates. For the assay, the culture medium was aspirated, and the wells were washed once with 2 mL of I-MEM. Subsequently, iDisper solutions (0.5 mL) containing papain at different concentrations (1, 2, or 4 mg/mL) or the control reagent TrypLE Express were added to each well. Plates were incubated at 37°C, and cell detachment was monitored and imaged at 0, 5, and 10 min using a phase-contrast microscope. After the final point, I-MEM (2.5 mL) was added, and the cells were thoroughly suspended by pipetting. The cell suspension was transferred to a 15 mL tube and centrifuged at 300 × g for 3 min at room temperature.

**Response 3-3:** Thank you for pointing this out. To clarify the embryo incubation conditions, we have explicitly added the humidity control details (maintained at 60% humidity) along with the phrase "(containing embryo tissue)"
**Revised text (Page 5, Line 82)**: Primary cells were isolated from the duck liver and chicken muscle. Fertilized duck eggs were purchased from Shiina Hatchery (Japan), and fertilized chicken eggs were obtained from Yamagishi (Wakayama, Japan). **Both types of eggs (containing embryo tissue) were incubated in a P-008 incubator (Showa Furanki, Saitama, Japan) maintained at 37 °C and 60% humidity**. The incubation periods were set to 14 and 12 days for the duck and chicken eggs, respectively, to ensure appropriate embryonic development.

**Comment 4:** *Results: In Fig 2d, quantify "4.1% failure rate" derivation (e.g., n= total cultures). Move S1 Appendix gene expression (S1 Fig) to main text as it supports phenotypic stability claims.*

**Response 4:** We appreciate the reviewer’s feedback. Following the suggestion, we moved the gene expression data from the S1 Appendix (formerly S1 Fig) to the main text as the new Fig 2 to support our phenotypic stability claims. Consequently, the original Fig 2 and Fig 3 were renumbered to Fig 3 and Fig 4, and the original S2 Fig–S6 Fig were renumbered to S1 Fig–S5 Fig. Additionally, we have revised the caption for the new Fig 3d to explicitly state the derivation of the 4.1% failure rate, including the total sample sizes (*n*) for each passage.
**Revised caption text (Page 13, Line 201):** The iDisper performance was evaluated using an industrially relevant cell population. We used primary duck liver-derived cells, which are used as raw materials for cell-based food. Under standard culture conditions, these cells proliferated as adherent cells and were predominantly mesenchymal cells. To evaluate the performance of the pre-culture step on cell quality, we compared gene expression profiles in cells at an early stage of passage (P0) and late passages (P2 and P6) (Fig 2). As a result, the expression of ALB and HNF4A, which are hepatocyte maturation markers [22], was significantly decreased in P2 and P6 cells compared to that in P0 cells. In addition, the expression levels of COL3A1 and DCN, which are highly expressed in mesenchymal cells [23], were significantly increased. Hepatocytes generally have a low proliferation profile [24]. This suggests that a cell population with a higher ability to adhere to the planar substrate and that is suitable for proliferation may have been selected. This indicated that the characteristics of the cells selected in the standard 2D cell culture were maintained.
**Revised caption text (Page 15, Line 255):** (d) Success rates for cell expansion from passage 0 (P0) to P2. Pie charts show the proportion of successful cultures versus those lost to culture failure or microbial contamination. The percentages of culture outcomes were calculated by dividing the number of specific events by the total number of evaluated cultures (*n*) for each passage; the total sample sizes are *n* = 219 for P0 (comprising 207 successful, 9 contaminations, and 3 failures), *n* = 511 for P1 (505 successful, and 6 failures), and *n* = 384 for P2 (379 successful, and 5 failures).

**Comment 5:** *Discussion: Briefly address scalability for powder formulation (mentioned but not quantified) and potential mammalian cell testing limitations. Compare allergen management to FDA consultations (refs 11-15).*

**Response 5**: We sincerely thank the reviewer for these constructive suggestions. We have revised the Discussion section to address all three points: First, we added a sentence acknowledging the scalability limits of our powder formulation. Regarding the lack of quantification, evaluating industrial scalability requires manufacturing trials on the scale of hundreds of kilograms. Providing such precise data is beyond the scope of this initial study, and we kindly ask for your understanding on this point. Second, we explicitly noted the lack of mammalian cell testing as a limitation of this study. Finally, as requested, we added a brief comparison of our allergen management approach to recent FDA pre-market consultations
**Revised text (Page 16, Line 272):** The upstream development process proposed in this study must be integrated with the downstream development process to bring products to market. In a previous study, we established a heat-based sterilization process. [20] However, as iDisper's core component, papain, is heat-sensitive, an industrial-scale product is likely to be a powder. This is a common formulation for food ingredients such as seasonings or powdered soup bases. Future developments must address key manufacturing parameters for powders, such as controlling the particle size to ensure product uniformity, solubility, and stability. [26, 27] Currently, the scalability of this powder formulation is limited to laboratory-scale proof-of-concept, necessitating future process engineering to address challenges such as homogeneous mass-blending and moisture control during large-scale storage.
**Revised text (Page 14, Line 233):** To evaluate its broader utility, we tested the iDisper on other avian cell types, including primary chicken muscle cells and DF-1. The iDisper achieved robust cell numbers and high viability across all cell types (Fig 4a). The product showed excellent storage stability, with no performance loss after 8 months at −20 °C (Fig 4b). The detachment performance decreased to approximately 30% after 12 months (S5 Fig). However, a limitation of the current study is that the dissociation efficacy of iDisper was exclusively validated using avian cells. Given that bovine and porcine species are next targets for the cell-based food industry, future studies should validate this versatility across a broad range of industrially relevant species, including mammalian and aquatic cells.
**Revised text (Page 16, Line 286):** By contrast, our framework yields products with visible, manageable risks. The complete composition and our proposed labeling, conceptually based on Japanese food labeling standards, are shown in (Supporting Information; S1 Table). When compared to recent FDA pre-market consultations for cell-cultured meat [13-17]—which rigorously evaluate whether allergenic processing aids remain in the final product—our approach similarly ensures safety by transparently declaring known allergens (e.g., "Contains enzymes derived from papaya")

**Comment 6:** *Figures/Tables: Ensure all scale bars are uniform (e.g., Fig 2a: 0.5 mm). Table 1 legend could note "Materials selected" more prominently.*

**Response 6**: We appreciate the reviewer’s feedback. We have revised the figures and tables to improve clarity and consistency as suggested.

1. **Figures:** All scale bars in the images (including Fig 3a: old Fig 2a) have been standardized to **0.5 mm** for uniformity.
2. **Table 1:** To make the legend more prominent, we have changed the phrase **"Materials selected"** to **boldface with an underline,** and increased its font size by **1.0 point**. These changes ensure that the key information is more easily identifiable for the readers.

Thank you for allowing us to strengthen our manuscript with your valuable comments and queries. We hope that these revisions are sufficient to make our manuscript suitable for publication in *PLOS One*, and look forward to hearing from you.

Yours sincerely,

Ikko Kawashima, PhD,

CTO
IntegriCulture Inc.
Email: [ikko@integriculture.com](mailto:ikko@integriculture.com)
Phone: +81 50 5470 9432
